# Economic Evaluation of Wastewater Surveillance Combined with Clinical COVID-19 Screening Tests, Japan

**DOI:** 10.3201/eid2908.221775

**Published:** 2023-08

**Authors:** Byung-Kwang Yoo, Ryo Iwamoto, Ungil Chung, Tomoko Sasaki, Masaaki Kitajima

**Affiliations:** Waseda University, Saitama, Japan (B.-K. Yoo);; Kanagawa University of Human Services, Kanagawa, Japan (B.-K. Yoo, U. Chung);; Shionogi & Co. Ltd. and AdvanSentinal Inc., Osaka, Japan (R. Iwamoto);; The University of Tokyo, Tokyo, Japan (U. Chung);; Independent consultant, Shiga, Japan (T. Sasaki); Hokkaido University, Hokkaido, Japan (M. Kitajima)

**Keywords:** COVID-19, respiratory infections, severe acute respiratory syndrome coronavirus 2, SARS-CoV-2, SARS, coronavirus disease, zoonoses, viruses, coronavirus, wastewater surveillance, screening, economic evaluation, economic efficiency, cost benefit analysis, return on investment, ROI, benefit to cost ratio, BCR, Japan

## Abstract

The COVID-19 pandemic has imposed substantial burdens on the global society. To find an optimal combination of wastewater surveillance and clinical testing for tracking COVID-19, we evaluated the economic efficiency of hypothetical screening options at a single facility in Japan. To conduct cost-benefit analyses, we developed standard decision models in which we assumed model parameters from literature and primary data, such as screening policies used at the Tokyo Olympic and Paralympic Village in 2021. We compared hypothetical 2-step screening options that used clinical PCR to diagnose COVID-19 after a positive result from primary screening using antigen tests (option 1) or wastewater surveillance (option 2). Our simulation results indicated that option 2 likely would be economically more justifiable than option 1, particularly at lower incidence levels. Our findings could help justify and promote the use of wastewater surveillance as a primary screening at a facility level for COVID-19 and other infectious diseases.

COVID-19, caused by SARS-CoV-2, has imposed substantial disease and social burdens on the global society; ≈6.85 million deaths were confirmed worldwide by February 2023 (*1*). To reduce disease burden, both clinical screening tests and epidemic surveillance systems are required and need to be efficiently implemented under tight budget constraints.

Although clinical PCR and antigen tests are essential for detecting individual cases, those tests have multiple limitations, such as testing avoidance behaviors, low detection rates among asymptomatic persons, and challenges when high demand for testing during epidemic peaks exceeds laboratory capacity. An additional limitation is the relatively high cost at a population level, which hinders frequent implementation even among high-risk subpopulations and essential workers. Because of those limitations, an epidemic surveillance system based on clinical tests tends to underestimate prevalence and have reduced representation because of insufficient sample sizes.

Wastewater surveillance is expected to address limitations of clinical tests (*2*). A sample of wastewater can be highly representative for all residents at a specific facility or for hundreds of thousands of residents in an area covered by a single wastewater treatment plant. Although wastewater surveillance is a risk measure of a community and not an individual resident, when compared as separate options, a simple cost comparison favors wastewater surveillance over clinical tests (*3*).

The appropriate sampling site can differ depending on the population level targeted by wastewater surveillance. When a large population is targeted, such as all residents within a citywide sewershed, sampling of influent wastewater at a wastewater treatment plant is most effective (*4*). When neighborhood-scale sewersheds are targeted, wastewater should be sampled from manholes or pumping stations (*5*). Finally, when a single facility is targeted, wastewater samples must be collected immediately after being discharged from the facility; in most cases, such samples can be collected from a manhole (*6*).

We aimed to find an optimal combination of wastewater surveillance and clinical testing that complement, rather than substitute for, each other. Therefore, we performed an economic evaluation to estimate the return on investment (ROI) of hypothetical screening options at a single facility in Japan.

## Methods

We conducted a cost-benefit analysis to estimate the economic efficiency of various hypothetical screening options for confirming SARS-CoV-2 infections among asymptomatic or presymptomatic persons at a single residential facility, as measured by ROI, an equivalent to benefit-to-cost ratio. If 1 option is cost-saving compared with its comparator, that option’s ROI is estimated to be >1. For example, an estimated ROI of 1.50 indicates that a $100 investment in 1 option will produce a net savings of $50. Our cost-benefit analyses adopted a societal perspective with a 1-month timeframe.

We compared 2 hypothetical 2-step screening options that used clinical PCR tests to diagnose individual COVID-19 cases after a positive result from a primary screening with antigen tests (option 1) or wastewater surveillance (option 2). Those screening options partly followed those used in the Tokyo Olympic and Paralympic Village in 2021 (*6*,*7*). We assumed antigen test results would be available in <1 hour, PCR test results would be available on the same day, and wastewater surveillance results would be available by the day after sampling.

More specifically, under option 1, the residents at a facility would all undergo antigen testing daily for 4 days as a primary screening. Any resident who tests positive would receive secondary screening on the same day with 2 PCR tests to confirm the diagnosis. Option 2 was to conduct wastewater surveillance at a facility as a primary screening for days 1–3. If a previous day’s wastewater surveillance indicated a positive result, all persons at the facility would undergo secondary screening with 2 consecutive PCR tests to clinically diagnose an infected case during days 2–4.

Option 1 and option 2 are substitutes only in terms of their primary screening, either antigen tests or facility-based wastewater surveillance. For both options, the primary screening (antigen tests or facility-based wastewater surveillance) and secondary screening (PCR for clinical diagnosis) are complementary.

We assumed model parameters on the basis of available literature and primary data and developed a standard decision model (Table 1; Appendix Figure 1). Our base-case analysis with a deterministic model assumed a point estimate for each parameter. To address the uncertainties of model parameters, we also implemented a probabilistic analysis with Monte Carlo simulations by assigning distributions (Table 1). For instance, we assumed a triangular distribution for the parameter sensitivity of wastewater surveillance using a mode of 66% (range 46%–84%). That parameter sensitivity could be affected by various factors, including variability in viral shedding over the course of an infection and between different infected persons, dilution and decay of virus in the sewer, and analytical sensitivity of the method used for virus detection in wastewater. Monte Carlo simulations provided the mean and the 95% probabilistic confidence interval (PCI) values of the ROI estimates. We used TreeAge software (https://treeage.com) to perform analyses for decision models.

**Table 1:** Decision model parameters in an economic evaluation of wastewater surveillance combined with clinical COVID-19 screening tests, Japan*

Parameters†	Point estimate (range)	Reference
Test characteristics		
Sensitivity		
Wastewater surveillance	0.66 (0.46–0.84)	M. Kitajima, unpub. data
PCR‡	0.74 (0.64–0.83)	(*8**,**9*)
Ratio of antigen test against PCR test	0.76 (0.54–0.97)	(*8**–**10*)
PCR test after positive antigen test	0.99 (0.64–0.999)	(*8**,**9*)
Specificity		
PCR	0.974 (0.96–0.995)	(*9**,**10*)
Antigen test	0.99 (0.97–0.995)	(*10*)
Ratio of wastewater surveillance against PCR test	0.99	
Cost		
Laboratory cost of wastewater surveillance per facility per day	$379 ($189–$758)	(*11**,**12*)
Labor cost to sample at a facility per facility per day	$1,136 ($152–$2,045)	(*13*)
Antigen test§	$16 ($10–$23)	(*14**,**15*)
Clinical PCR§	$38 ($20–$53)	(*14**,**15*)
Isolation per test-positive case	$758 ($379–$1,515)	(*16*)
Hospitalization per case¶	$19,394 ($16,212–$25,227)	(*17**–**19*)
Value of QALY saved per case	$37,879	(*20*)
Other		
Incidence per day per 1 million residents	100 (10–10,000)	(*4**,**21*)
No. residents at a facility	100 (50–200)	(*6**,**22*)
Mortality rate among persons who test positive#	0.0035 (0.0018–0.0104)	(*23*)
Ratio of mortality rate among persons >80 years of age vs. general population	19 (15–22)	(*23*)
Life-years saved by avoiding COVID-19	11.4 (11.1–11.7)	(*24**,**25*)
Ratio to convert life-years saved to QALYs saved	0.68 (0.64–0.71)	(*24**,**26*)
Hospitalization rate among persons who test positive	0.18 (0.04–0.40)	(*17*)
Proportion of severe cases among hospitalized cases	0.1 (0.05–0.19)	(*17*)
Effective reproduction number of infected cases	1.3 (0.9–2.0)	(*27*)
Screening effectiveness in reducing hospitalization and mortality rates because of an earlier diagnosis	0.54 (0.23–0.62)	(*28*)
Ratio of loss value of missing an infected case compared with benefit value of finding an infected case	1 (0–2.0)	

*All monetary values are expressed in 2022 US dollars. QALY, quality-adjusted life-years. †All parameters with a minimum and a maximum value in this table are defined as a triangular distribution in the probabilistic analysis, detailed in the Appendix (https://wwwnc.cdc.gov/EID/article/29/8/22-1775-App1.pdf). ‡Because the second clinical PCR test was conducted immediately after the first clinical PCR test, the sensitivity of the second clinical PCR was assumed to be equal to the specificity of the PCR test in this table. §Test cost plus labor cost for sampling; 30 min multiplied by minimum wage of $7 USD per hour (*15*). ¶Hospitalization cost was assumed as $16,212 + $64,394 x (proportion of severe cases among hospitalized cases – 0.05). #The range was defined to range from the rate before the vaccination period to the rate after the vaccination period (Appendix).

Because economic efficiency is highly sensitive to the disease incidence, our base-case analysis included the 3 scenarios: 10, 100, or 1,000 newly reported clinically positive cases per million residents per day (PMPD) in the area around the facility. In other words, our study did not assign a certain distribution for the incidence because of a very wide range of feasible values.

The 10 PMPD incidence value corresponds to the minimum level at which wastewater surveillance sampled at a wastewater treatment plant can detect SARS-CoV-2 (*4*). Our 1-way sensitivity analyses all assumed the incidence value of 100 PMPD, above which a correlation was observed in our primary data between SARS-CoV-2 RNA load in wastewater sampled at a wastewater treatment plant and the incidence based on clinical PCR tests in the area (*21*).

The 1,000 PMPD incidence value is equal to the ratio of 1 newly infected case among 1,000 residents in a hypothetical facility, and 1,000 was close to the smallest population of the sampling area in the Tokyo Olympic and Paralympic Village in 2021 (*6*). Our base-case analyses all assumed the facility had 100 residents, which we based on the average number of beds in long-term care facilities (LTCFs) in Japan (*22*).

Hypothetical study populations in our base-case analyses all were 100 residents at a LTCF who were expected to receive greater benefits from screening tests in terms of preventing COVID-19–related illness and death compared with the general population. For instance, LTCF residents in Japan have an average age of ≈86 years (*29*) and were estimated to be 19 times as likely to die after a clinical COVID-19 diagnosis than the general population in Japan (*23*).

Our study estimated the benefit of confirming 1 infected case by PCR under each screening option by using 2 components: the benefit of reducing hospitalization and death for a confirmed case, and the benefit of preventing secondary infection. Because scant literature addressed the effectiveness of screening in reducing hospitalization and mortality rates among persons who test positive, we assumed effectiveness was equivalent to the clinical efficacy of antiviral agents among patients with COVID-19 at its early stage (e.g., <7 days after the onset of signs or symptoms), who are not hospitalized yet but could be subsequently hospitalized or die (*28*). We reduced the clinical efficacy by 30%, because 30% of infected persons never develop symptoms (*30*). Consequently, our screening effectiveness had a triangular distribution with a mode of 0.54 (range 0.23–0.62). We estimated the benefit of preventing secondary infection to be 0.57 under our base-case analyses, which was dependent on a reproduction number of 1.3 (*27*), an infectious period of 8.03 days (*31*), and other factors (*30*). In addition, our model accounted for the loss of missing an infected case that produced a second-generation infected case every day (Appendix).

To assign benefit values for reducing hospitalization and mortality rates, we estimated the related monetary value for 3 outcomes among confirmed cases: isolation (*14*–*16*), hospitalization (*17*–*19*), and death (*20*,*24*–*26*). All monetary values are expressed in 2022 US dollars (USD). We assigned a value for death by applying the monetary value of $37,879 for each quality-adjusted life-year (QALY) saved or lost under the cost-effectiveness analysis set by the Ministry of Health, Labour and Welfare of Japan (*20*). To estimate QALYs lost due to COVID-19, we first calculated life years lost based on age at death (*24*) and life expectancy among a certain age and sex (*25*). To convert life years lost to QALYs lost, we applied the ratios estimated among the population of the Netherlands (*26*), because the relevant data were not available for Japan.

## Results

When COVID-19 incidence was 10 PMPD, our deterministic base-case analysis indicated that option 1 alone, compared with doing nothing (comparator do-nothing), was not economically justifiable because its cost ($67.04) exceeded its benefit ($1.39) and the ROI of 0.021 ($1.39/$67.04) was <1.0 (Table 2). Although option 2 alone compared with do-nothing was not justifiable because of the low ROI (0.021), option 2 became justifiable when its comparator was changed from do-nothing to option 1. That is, compared with option 1, option 2 saved $13.44, which could be interpreted as relative benefit, and had a $0.25 lower benefit, which could be interpreted as relative cost. Thus, compared with option 1, the relative value of option 2 was a high ROI of 54 ($13.44/$0.25) (Table 2).

**Table 2:** Base-case analysis with a deterministic model in an economic evaluation of wastewater surveillance combined with clinical COVID-19 screening tests, Japan*

**Table 2. **Base-case analysis with a deterministic model in an economic evaluation of wastewater surveillance combined with clinical COVID-19 screening tests, Japan*											
Incidence†	Option 1				Option 2				Relative value of option 2		
	Cost	Benefit	ROI‡		Cost	Benefit	ROI‡		Incremental cost§	Incremental benefit¶	Relative ROI#
10	$67.04	$1.39	0.021		$53.60	$1.14	0.021	–$13.44	–$0.25	54
100	$67.05	$14.09	0.21		$53.61	$11.94	0.22		–$13.43	–$2.15	6.25
1,000	$67.12	$141.11	2.10		$53.75	$119.94	2.23		–$13.37	–$21.16	0.63

*Option 1 is clinical testing only; option 2 is wastewater surveillance and clinical testing. If one option is cost-saving compared with its comparator, the option’s ROI is estimated to exceed 1. The comparator of options 1 and 2 is do-nothing. All monetary values are expressed in 2022 US dollars (USD). ROI, return on investment. †Disease incidence per day per 1 million residents in the area. ‡ROI is benefit divided by cost for each option. §Incremental cost is the cost of option 2 minus cost of option 1. A negative value of incremental cost indicates that option 2 has a lower cost or is cost-saving, compared with option 1. This could be interpreted as option 2’s relative benefit. ¶Incremental benefit is the benefit of option 2 minus benefit of option 1. A negative value of incremental benefit indicates that option 2 has a lower benefit compared with option 1, which could be interpreted as option 2’s relative cost. #Relative ROI is incremental cost divided by incremental benefit.

When COVID-19 incidence was 1,000 PMPD under our base-case analysis, we estimated the ROI of option 1 to be 2.10 and of option 2 to be 2.23 (Table 2). One-way sensitivity analysis of the deterministic model showed the threshold incidence values, above or below which an option’s ROI is >1. Those threshold values were 480 PMPD for option 1 alone, 450 PMPD for option 2 alone, and 630 PMPD for the relative value of option 2 (Table 3). One-way sensitivity analysis also showed that when incidence increased, the ROI of options 1 and 2 increased and that the relative value of option 2 declined (Figure).

**Table 3:** One-way sensitivity analyses of the base-case analysis of the incidence parameter in an economic evaluation of wastewater surveillance combined with clinical COVID-19 screening tests, Japan*

Incidence†									Relative value of option 2		
	Option 1				Option 2				Incremental cost§	Incremental benefit¶	Relative ROI#
	Cost	Benefit	ROI‡		Cost	Benefit	ROI‡				
10	$67.04	$1.39	0.02		$53.60	$1.14	0.02		–$13.44	–$0.25	54
50	$67.04	$7.03	0.10		$53.61	$5.94	0.11		–$13.44	–$1.09	12
100	$67.05	$14.09	0.21		$53.61	$11.94	0.22		–$13.43	–$2.15	6
400	$67.07	$56.43	0.84		$53.66	$47.94	0.89		–$13.41	–$8.49	1.58
445	$67.08	$62.78	0.94		$53.67	$53.34	0.99		–$13.41	–$9.44	1.42
450	$67.08	$63.49	0.95		$53.67	$53.94	1.01		–$13.41	–$9.54	1.40
475	$67.08	$67.01	0.999		$53.67	$56.94	1.06		–$13.41	–$10.07	1.33
480	$67.08	$67.72	1.010		$53.67	$57.54	1.07		–$13.41	–$10.18	1.32
500	$67.08	$70.54	1.05		$53.68	$59.94	1.12		–$13.40	–$10.60	1.26
600	$67.09	$84.65	1.26		$53.69	$71.94	1.34		–$13.40	–$12.71	1.05
630	$67.09	$88.89	1.32		$53.70	$75.54	1.41		–$13.39	–$13.35	1.004
635	$67.09	$89.59	1.34		$53.70	$76.14	1.42		–$13.39	–$13.45	0.996
700	$67.10	$98.77	1.47		$53.71	$83.94	1.56		–$13.39	–$14.83	0.90
1,000	$67.12	$141.11	2		$53.75	$119.94	2		–$13.37	–$21.16	0.63
2,000	$67.20	$282.23	4		$53.91	$239.95	4		–$13.29	–$42.29	0.31
5,000	$67.45	$705.62	10		$54.37	$599.96	11		–$13.07	–$105.66	0.12
10,000	$67.85	$1,411.26	21		$55.14	$1,199.97	22		–$12.71	–$211.29	0.06

*Option 1 is clinical tests only; option 2 is wastewater surveillance and clinical tests. If an option is cost-saving compared with its comparator, the option’s ROI is estimated to exceed 1. The comparator of options 1 and 2 is do-nothing. All monetary values were expressed in 2022 US dollars. ROI, return on investment. †Disease incidence per day per 1 million residents in the area. ‡ROI is benefit divided by cost for each option. §Incremental cost is the cost of option 2 minus cost of option 1. A negative value of incremental cost indicates that option 2 has a lower cost or is cost-saving, compared with option 1. This could be interpreted as option 2’s relative benefit. ¶Incremental benefit is the benefit of option 2 minus benefit of option 1. A negative value of incremental benefit indicates that option 2 has a lower benefit compared with option 1, which could be interpreted as option 2’s relative cost. #Relative ROI is incremental cost divided by incremental benefit.

**Figure:**
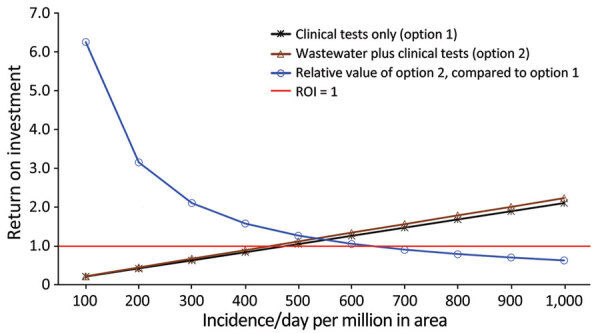
ROI comparison of 2 options used in an economic evaluation of wastewater surveillance combined with clinical COVID-19 screening tests, Japan. ROIs for the relative value of option 2 are expressed as log_10_ and determined by 1-way sensitivity analyses of the base-case analysis (Table 3). Red horizontal line indicates ROI = 1. ROI, return on investment.

Additional 1-way sensitivity analyses of the base-case analysis showed that within the feasible range of parameters, all 3 types of ROI estimates were sensitive to incidence and had values above and below 1.0. The ROI estimates of options 1 and 2 alone, compared with do-nothing, were robust to all parameters except incidence. The ROI estimates of options 1 and 2 alone had a negative association with test costs and a positive association with test sensitivity and specificity (Table 4).

**Table 4:** One-way sensitivity analyses of the base-case analysis in an economic evaluation of wastewater surveillance combined with clinical COVID-19 screening tests, Japan*

Parameters	Parameter values	ROI†		Relative ROI‡
		Option 1	Option 2	
Test characteristics				
Sensitivity				
Wastewater surveillance	0.46	0.21	0.12	2
	0.84	0.21	0.26	80
PCR	0.64	0.18	0.22	32
	0.83	0.23	0.22	4
Ratio of antigen test against PCR test	0.54	0.14	0.22	−5.43
	0.97	0.24	0.22	3
PCR test after positive antigen test	0.64	0.12	0.10	5
	0.999	0.21	0.22	6
Specificity				
PCR	0.96	0.21	0.21	5
	0.995	0.21	0.25	9
Antigen test	0.97	0.19	0.22	9
	0.995	0.22	0.22	6
Cost				
Wastewater surveillance cost per day per facility				
Laboratory cost	$189	0.21	0.25	9
	$758	0.21	0.18	0.96
Labor cost to sample	$152	0.21	0.50	20
	$2,045	0.21	0.15	−6.43
Antigen test¶	$10	0.33	0.22	−4.91
	$23	0.15	0.22	19
Clinical PCR¶	$20	0.215	0.24	7
	$53	0.207	0.21	5
Isolation per test-positive case	$379	0.209	0.222	6.33
	$1,515	0.212	0.224	6.09
Hospitalization per case#	$16,212	0.18	0.19	7
	$25,227	0.26	0.27	5
Other				
Incidence per day per 1 million population	10	0.02	0.02	54
	10,000	21	22	0.06
No. residents at a facility	50	0.21	0.12	−14.89
	1,000	0.21	0.94	25
Mortality rate among persons who test positive	0.0018	0.19	0.20	7
	0.0104	0.30	0.32	4
Ratio of mortality rate among persons >80 years of age vs. the general population	0.5	0.004	0.004	161
	22	0.24	0.26	5
Life-years saved by avoiding COVID-19	11.1	0.209	0.221	6.28
	11.7	0.211	0.224	6.21
Ratio to convert life-years saved to QALYs saved	0.64	0.207	0.220	6.33
	0.71	0.212	0.225	6.19
Hospitalization rate among persons who test positive	0.04	0.08	0.08	14
	0.40	0.42	0.46	4
Proportion of severe cases among hospitalized cases	0.05	0.18	0.19	7
	0.19	0.26	0.28	5
Effective reproduction number of infected cases	0.9	0.28	0.31	7
	2.0	0.17	0.16	5
Effectiveness of screening in reducing hospitalization and mortality rates because of an earlier diagnosis	0.23	0.09	0.10	14
	0.62	0.24	0.26	5
Ratio of loss value of missing and infected cases compared with benefit value of finding an infected case	0.0	0.25	0.31	148
	2.0	0.17	0.13	3

*The lower and upper bounds of each parameter are shown to illustration the association between a parameter and its ROI. Incidence was assumed to be 100 persons per day per 1 million residents in the area. All monetary values are expressed in 2022 US dollars. QALY, quality-adjusted life-years; ROI, return on investment. †Option 1 is clinical tests only; option 2 is wastewater surveillance and clinical tests. ‡Relative ROI of option 2 compared with option 1.

The estimated range of the ROI for the relative value of option 2 includes negative values (Table 4). For the ratio of sensitivity of antigen tests against PCR, option 2 was always preferred over option 1; option 2 dominated option 1 when the ROI estimates were negative for that ratio. In other words, a simple linear relationship did not occur between the ratio of sensitivity of antigen tests against PCR and the ROI for the relative value of option 2. For instance, when that ratio increased from 0.64 to 0.97, the ROI estimate for the relative value of option 2 was always >1 (Appendix Table 8). When the ratio was 0.638, option 2’s benefit became equal to option 1’s benefit, which did not mathematically enable estimation of the ROI for the relative value of option 2. When the ratio increased from 0.54 to 0.63, option 2’s benefit exceeded option 1’s benefit; thus, option 2 dominated option 1.

The ROI estimates regarding the relative value of option 2 were sensitive to 3 cost-related parameters. In other words, an estimated threshold point existed, below or above which a preferred option changed. For instance, option 2 was preferred only when the labor cost to sample a facility was lower than the threshold point of $1,512. When labor cost exceeded that threshold point, option 1 was preferred. Likewise, when the cost of the antigen test was lower than the threshold point of $13.18, option 1 was preferred, but when it was greater than that threshold point, option 2 was preferred. Because the cost of wastewater surveillance per facility was fixed, the cost per facility resident could be substantially reduced by a larger number of facility residents. Therefore, when the number of residents was lower than the threshold point of 81, option 1 was preferred, but when it was greater than that threshold, option 2 was preferred.

The probabilistic analyses showed that the base-case analyses with a deterministic model were robust, particularly for cost, benefit, and ROI estimates for option 1 alone or option 2 alone (Table 5). Although the estimated PCIs included a large negative value as a lower bound, option 2 was mostly preferred to option 1 when the incidence was 10 or 100 PMPD. More specifically, over 1,000 iterations, when incidence was 10 PMPD, option 2 was preferred in 84.7% of the time; when incidence was 100 PMPD, option 2 was preferred 80.8% of the time; and when incidence was 1,000 PMPD, option 2 was preferred 25.2% of the time. Thus, qualitative conclusions of probabilistic analyses were similar to those of deterministic analyses.

**Table 5:** Base-case analysis using a deterministic model and a probabilistic model in an economic evaluation of wastewater surveillance combined with clinical COVID-19 screening tests, Japan*

Incidence†	Option 1				Option 2				Relative value of option 2		
	Cost	Benefit	ROI 1‡		Cost	Benefit	ROI 2‡		Inc. cost§	Inc. benefit¶	Rel. ROI#
10											
DA	$67.04	$1.39	0.021		$53.60	$1.14	0.021		–$13.44	–$0.25	54
Mean PA (95% PCI)	$70.03 ($49.85–$90.25)	$1.43 ($0.42–$2.85)	0.021 (0.006–0.043)		$50.68 ($25.27–$90.23)	$0.97 ($0.19–$2.04)	0.021 (0.004–0.051)		–$19.35 (–$54.48 to $24.31)	–$0.46 (–$1.20 to $0.08)	45 (−194 to 387)
100											
DA	$67.05	$14.09	0.21		$53.61	$11.94	0.22		–$13.43	–$2.15	6.25
Mean PA (95% PCI)	$68.54 ($48.77–$88.86)	$14.75 ($5.11–$28.35)	0.22 (0.07–0.45)		$50.86 ($24.95–$92.14)	$10.37 ($3.03–$20.71)	0.23 (0.05–0.60)		–$17.68 (–$52.33 to $23.34)	–$4.38 (–$11.35 to $1.31)	5.74 (−24 to 37)
1,000											
DA	$67.12	$141.11	2.10		$53.75	$119.94	2.23		–$13.37	–$21.16	0.63
Mean PA (95% PCI)	$69.50 ($48.76–$89.54)	$147.29 ($52.37– $279.00)	2.17 (0.73–4.57)		$50.61 ($24.56–$89.89)	$104.58 ($30.91–$215.00)	2.29 (0.55–5.59)		–$18.89 (–$52.28 to $23.15)	–$42.71 (–$110 to $8.65)	0.34 (−2.14 to 3.71)

*A probabilistic model to compare clinical tests only (option 1) to wastewater surveillance combined with clinical tests (option 2). If one option is cost-saving compared with its comparator, the option’s ROI is estimated to exceed 1. The comparator of options 1 and 2 is do-nothing. DA, deterministic model analysis; inc., incremental; PA, probabilistic model analysis with Monte Carlo simulations; PCI, probabilistic confidence interval; rel., relative; ROI, return on investment. †Disease incidence per day per 1 million residents in the area. ‡ROI is benefit divided by cost for each option. §Incremental cost is the cost of option 2 minus cost of option 1. A negative value of incremental cost indicates that option 2 has a lower cost or is cost-saving, compared with option 1. This could be interpreted as option 2’s relative benefit. ¶Incremental benefit is the benefit of option 2 minus benefit of option 1. A negative value of incremental benefit indicates that option 2 has a lower benefit compared with option 1, which could be interpreted as option 2’s relative cost. #Relative ROI is incremental cost divided by incremental benefit.

## Discussion

Our simulation results indicate that a primary screening with wastewater surveillance (option 2) at a single facility was highly likely to be economically more justifiable than a primary screening with antigen tests (option 1), particularly at lower incidence levels (<630 PMPD). Option 2 tended to have a much lower cost (interpreted as relative benefit) and a slightly lower benefit (interpreted as relative cost) compared with option 1. Of note, when the comparator was do-nothing, option 1 alone and option 2 alone had low economic efficiency when the disease incidence was low; option 1 alone was economically justifiable only when the incidence was >480 PMPD and option 2 alone was economically justifiable only when the incidence was >450 PMPD. At incidence levels >1,000 PMPD, option 2 is economically less efficient than option 1 because clinical tests would not be implemented on day 1 under option 2, which would lead to more secondary infections and more costs for isolation or hospitalization. Our results appeared generally robust to the feasible range of model parameters, although some results were sensitive to parameters related to the disease incidence and cost of tests.

Our analytical models are expected to have high generalizability to and be robust for SARS-CoV-2 variants, unlike vaccination effectiveness, which can potentially be reduced by variants. In addition, our analytic approach would be readily applicable to other emerging infectious diseases.

The negative ROI estimates regarding the relative value of option 2 should be interpreted with caution because 2 opposite interpretations are possible. One interpretation prefers option 2, such as when option 2 detected many more infected cases than option 1 at a facility with <77 residents. On the contrary, the other interpretation prefers option 1, such as when fewer COVID-19 cases were missed by option 1 than option 2 and when the antigen test cost was <$12.64.

We expected the face validity of our simulation results to be achieved to some extent, partly because the assumptions of our hypothetical screening options mainly followed the screening policies used in the Tokyo Olympic and Paralympic Village (*6*,*7*). Also, the assumed range of the laboratory cost for wastewater surveillance ($189–$758) appeared reasonable, compared with costs reported by other studies (*11*,*12*,*32*). In addition, we used conservative assumptions in our base-case analysis, such as relatively high costs for additional labor to sample wastewater at a facility for surveillance (*13*). Another set of conservative assumptions that reduced the benefit of confirming 1 infected case were the exclusion of COVID-19–related medical expenditure for outpatient care and the possible financial loss related to shutdown of a LTCF. We excluded those items from our analyses because cost-related data were absent in the literature.

One weakness of this study is the limited generalizability to other settings. We assumed the monetary value of finding 1 COVID-19 case at a facility depended partly on related medical expenditure and QALY saved. QALY varies in different countries; in Japan, the value is $37,879/QALY (*20*). Also, the monetary value of finding 1 case consisted of mortality rate in the population, hospitalization rate in the population, and medical expenditures per hospitalized case, all of which could vary substantially at the population level because of viral variants occurring over time and across regions within a country. In addition, mortality and hospitalization rates vary markedly among subpopulations defined by age and high-risk chronic conditions. Such uncertainties indicate the need to frequently update the simulation model to correspond to regional epidemics and target populations.

Because of the absence of literature, the validity of our ROI estimates was difficult to compare with estimates from previous studies. Although 1 study compared wastewater surveillance at a treatment plant and clinical PCR tests in its costs, that study compared cost per population screened without accounting for clinically confirmed cases after wastewater surveillance (*3*). Therefore, the estimates in that study were not appropriate comparisons for our ROI estimates. When the goal of screening is to identify and isolate an infected case, wastewater surveillance should be used as a primary screening, after which secondary screening should be performed by using clinical tests.

Major policy implications derived from this study’s findings are exemplified by the threshold levels to start or suspend a specific screening option. Compared with do-nothing, threshold incidence levels were 480 PMPD for option 1 alone and 450 PMPD for option 2 alone, but those thresholds are <1,000 PMPD. The 1,000 PMPD incidence is equivalent to 1 newly infected case at a single large facility with 1,000 residents. That is, before finding the first newly infected case at a single facility, options 1 and 2 should be started, ideally triggered when the incidence of the area around the facility, such as the city, town, or neighborhood, reaches the threshold levels we reported for each option.

The ROI estimates for the relative value of option 2 compared with option 1 tended to be high at a very low incidence, when the absolute benefit of option 2 is small compared with do-nothing. One practical incidence level to trigger option 2 is 10 PMPD, above which wastewater surveillance conducted by using a recently developed method can detect SARS-CoV-2 RNA at a treatment plant (*4*). Another trigger incidence is 100 PMPD, above which conventional wastewater surveillance methods can detect SARS-CoV-2 RNA (*4*). Regularly monitoring data from wastewater surveillance at a treatment plant could enable efficient triggers for option 1 and option 2 at a specific facility in the same area.

Because wastewater surveillance at a treatment plant covers a city-scale population, the additional cost per resident would be very small, even when focusing on an institutionalized population; for instance, increasing the per resident cost in our model by <1%. Although the central government of Japan implemented pilot projects of wastewater surveillance at both city and facility levels during fiscal year 2022 (*33*), government officials did not expand the scale of those projects, partly because of a lack of evidence regarding economic efficiency. Thus, our findings could help the central government of Japan justify the expansion of these projects.

Another major policy implication is the threshold level for the number of residents at a facility. Our base-case analyses used hypothetical study populations of 100 residents at an LTCF. Our sensitivity analyses showed that the ROIs for option 2 alone and relative value of option 2 might increase when the number of residents increased; hence, wastewater surveillance cost per resident declined. The number of residents per facility could be easily increased to >1,000, the upper bound of our 1-way sensitivity analyses, if a facility, such as a large apartment complex, included younger residents. However, a lower mortality rate for younger residents would reduce the general screening benefit, thus reducing the ROI. An estimated minimum (threshold) number of 81 residents at an LTCF appears to help set a public guideline for wastewater surveillance.

Additional policy implications would help set goals for related industry. Because option 1 and option 2 differ in a primary screening, the difference in sensitivity between antigen tests and wastewater surveillance affected the economic efficiency for the relative value of option 2. Improved sensitivity of antigen tests is feasible but requires a longer time to diagnose a case, which reduces the benefit of antigen tests by postponing the diagnosis timing compared with 1-hour diagnosis time assumed under our base-case analysis. In other words, shortening the time to diagnosis for a screening test result would generally improve the test’s economic efficiency, a goal for related industry.

Future research could further explore the monetary values of time needed for screening, such as time required by caregivers who collect samples from LTC residents or young children. If those time costs are much larger in a certain setting, like a kindergarten, the relative economic efficiency of wastewater surveillance against clinical tests would increase.

Although one of the general advantages of wastewater surveillance is fewer privacy and stigmatization concerns than possible with clinical surveillance (*34*), ethical issues could arise in 2 cases. First, targeting a specific facility or a small catchment could lead to social harm and financial burdens to the targeted population (*34*). Second, regardless of the target population size, ethical issues might arise when the wastewater surveillance is used for applying restrictive measures, such as group quarantine or business closure in the target area or facility (*35*). Researchers, policymakers, and regulators need to collaborate to account for ethical issues in implementing wastewater surveillance (*36*), which could enable wastewater surveillance to represent a new frontier in surveillance, monitoring, and screening.

In conclusion, our findings could help justify and promote the use of wastewater surveillance as a primary screening at a single facility when a set of quantified conditions estimated in our simulation are met. Of note, regular wastewater surveillance at a treatment plant will help trigger the start of any screening tests at a specific facility. Because few economic evaluations of wastewater surveillance have yet been conducted, our findings can contribute to related academic fields and policy making.

Appendix Additional information on economic evaluation of wastewater surveillance combined with clinical COVID-19 screening tests, Japan.

## References

[1] World Health Organization. Coronavirus disease (COVID-19) pandemic [cited 2023 Feb 23]. https://www.who.int/emergencies/diseases/novel-coronavirus-2019

[2] Kitajima M, Ahmed W, Bibby K, Carducci A, Gerba CP, Hamilton KA, et al. SARS-CoV-2 in wastewater: State of the knowledge and research needs. Sci Total Environ. 2020;739:139076. 10.1016/j.scitotenv.2020.139076 32758929 PMC7191289

[3] Hart OE, Halden RU. Computational analysis of SARS-CoV-2/COVID-19 surveillance by wastewater-based epidemiology locally and globally: Feasibility, economy, opportunities and challenges. Sci Total Environ. 2020;730:138875. 10.1016/j.scitotenv.2020.138875 32371231 PMC7175865

[4] Ando H, Murakami M, Ahmed W, Iwamoto R, Okabe S, Kitajima M. Wastewater-based prediction of COVID-19 cases using a highly sensitive SARS-CoV-2 RNA detection method combined with mathematical modeling. Environ Int. 2023;173:107743. 10.1016/j.envint.2023.107743 36867995 PMC9824953

[5] Oh C, Zhou A, O’Brien K, Jamal Y, Wennerdahl H, Schmidt AR, et al. Application of neighborhood-scale wastewater-based epidemiology in low COVID-19 incidence situations. Sci Total Environ. 2022;852:158448. 10.1016/j.scitotenv.2022.158448 36063927 PMC9436825

[6] Kitajima M, Murakami M, Kadoya SS, Ando H, Kuroita T, Katayama H, et al. Association of SARS-CoV-2 Load in Wastewater With Reported COVID-19 Cases in the Tokyo 2020 Olympic and Paralympic Village From July to September 2021. JAMA Netw Open. 2022;5:e2226822. 10.1001/jamanetworkopen.2022.26822 35994292 PMC9396362

[7] Kitajima M, Murakami M, Iwamoto R, Katayama H, Imoto S. COVID-19 wastewater surveillance implemented in the Tokyo 2020 Olympic and Paralympic Village. J Travel Med. 2022;29:taac004. 10.1093/jtm/taac004 PMC9156005 35134222

[8] Marando M, Tamburello A, Gianella P, Taylor R, Bernasconi E, Fusi-Schmidhauser T. Diagnostic sensitivity of RT-PCR assays on nasopharyngeal specimens for detection of SARS-CoV-2 infection: A Systematic Review and Meta-Analysis. Caspian J Intern Med. 2022;13(Suppl 3):139–47. 35872685 10.22088/cjim.13.0.139 PMC9272971

[9] Tsang NNY, So HC, Ng KY, Cowling BJ, Leung GM, Ip DKM. Diagnostic performance of different sampling approaches for SARS-CoV-2 RT-PCR testing: a systematic review and meta-analysis. Lancet Infect Dis. 2021;21:1233–45. 10.1016/S1473-3099(21)00146-8 33857405 PMC8041361

[10] Tapari A, Braliou GG, Papaefthimiou M, Mavriki H, Kontou PI, Nikolopoulos GK, et al. Performance of antigen detection tests for SARS-CoV-2: a systematic review and meta-analysis. Diagnostics (Basel). 2022;12:1388. 10.3390/diagnostics12061388 35741198 PMC9221910

[11] Kantor RS, Greenwald HD, Kennedy LC, Hinkle A, Harris-Lovett S, Metzger M, et al. Operationalizing a routine wastewater monitoring laboratory for SARS-CoV-2. PLOS Water. 2022;1:e0000007. 10.1371/journal.pwat.0000007

[12] Safford HR, Shapiro K, Bischel HN. Opinion: Wastewater analysis can be a powerful public health tool-if it’s done sensibly. Proc Natl Acad Sci U S A. 2022;119:e2119600119. 10.1073/pnas.2119600119 35115406 PMC8833183

[13] Ministry of Land Infrastructure. Transport and Tourism. Engineer unit price for a design outsourcing for fiscal year 2021 [in Japanese] [cited 2022 Aug 23]. https://www.mlit.go.jp/tec/content/001387446.pdf

[14] Ministry of Health Labour and Welfare. Approval information for in-vitro diagnostic reagents (test kits) for COVID-19 [in Japanese] [cited 2022 Aug 22]. https://www.mhlw.go.jp/stf/newpage_11331.html

[15] Ministry of Health Labour and Welfare. Current status of minimum wage amendments by regions for FY 2021 [in Japanese] [cited 2022 Aug 4]. https://www.mhlw.go.jp/stf/seisakunitsuite/bunya/koyou_roudou/roudoukijun/minimumichiran

[16] Ministry of Health Labour and Welfare. Handling of the emergency comprehensive support program (for medical care) for COVID-19 in FY 2022 [in Japanese] [cited 2022 Aug 31]. https://www.mhlw.go.jp/content/000968054.pdf

[17] Ministry of Health Labour and Welfare. Survey on medical treatment and number of inpatient beds [in Japanese] [cited 2021 Sep 14]. https://www.mhlw.go.jp/stf/seisakunitsuite/newpage_00023.html

[18] Global Health Consulting. The unit price of a COVID-19 patient—54,000 yen for mild cases, 80,000 yen for moderate cases, and 142,000 yen for severe cases–is not worth the investment cost, according to the president of Japan Municipal Hospital Association (JMHA) [in Japanese] [cited 2021 Nov 24]. https://gemmed.ghc-j.com/?p=38100

[19] Ministry of Health Labour and Welfare. Amendments for the rules on “major medical institutions for COVID-19 and cooperative medical institutions accepting suspected COVID-19 patients” [in Japanese] [cited 2022 Aug 31]. https://www.mhlw.go.jp/content/000764832.pdf

[20] Central Social Insurance Medical Council, Ministry of Health, Labour and Welfare (MHLW). Examination of scientific matters for cost-effectiveness evaluation (part 4) [in Japanese] [cited 2022 Aug 31]. https://www.mhlw.go.jp/content/12404000/000380564.pdf

[21] Kanagawa Prefectural Government. Wastewater surveillance reports at the wastewater treatment plants in Kanagawa prefecture, Japan [cited 2022 Sep 2]. https://www.pref.kanagawa.jp/docs/ga4/covid19/simulation.html

[22] Ministry of Health Labour and Welfare. Overview of survey of institutions and establishments for long-term care for FY 2020 [in Japanese] [cited 2022 Aug 23]. https://www.mhlw.go.jp/toukei/saikin/hw/kaigo/service20/dl/kekka-gaiyou_1.pdf

[23] Ministry of Health Labour and Welfare. Visualizing the data: information on COVID-19 infections [in Japanese] [cited 2022 Jul 26]. https://covid19.mhlw.go.jp/extensions/public/index.html

[24] National Institute of Population and Social Security Research (IPSS). Data on COVID-19 [cited 2022 Aug 31]. www.ipss.go.jp/projects/j/Choju/covid19/index-en.asp

[25] Ministry of Health. Labour and Welfare. Abridged life tables for Japan 2020 [cited 2022 Aug 25]. https://www.mhlw.go.jp/english/database/db-hw/lifetb20/dl/lifetb20-06.pdf

[26] Wouterse B, Ram F, van Baal P. Quality-adjusted life-years lost due to COVID-19 mortality: methods and application for The Netherlands. Value Health. 2022;25:731–5. 10.1016/j.jval.2021.12.008 35500946 PMC8810280

[27] Neilan AM, Losina E, Bangs AC, Flanagan C, Panella C, Eskibozkurt GE, et al. Clinical impact, costs, and cost-effectiveness of expanded severe acute respiratory syndrome coronavirus 2 testing in Massachusetts. Clin Infect Dis. 2021;73:e2908–17. 10.1093/cid/ciaa1418 32945845 PMC7543346

[28] Lai CC, Wang YH, Chen KH, Chen CH, Wang CY. The clinical efficacy and safety of anti-viral agents for non-hospitalized patients with COVID-19: a systematic review and network meta-analysis of randomized controlled trials. Viruses. 2022;14:1706. 10.3390/v14081706 36016328 PMC9415971

[29] e-Stat. Survey of institutions and establishments for long-term care—result detail for FY 2019 [in Japanese] [cited 2022 Jun 16]. https://www.e-stat.go.jp/stat-search/files?&stat_infid=000032069585

[30] Johansson MA, Quandelacy TM, Kada S, Prasad PV, Steele M, Brooks JT, et al. SARS-CoV-2 transmission from people without COVID-19 symptoms. JAMA Netw Open. 2021;4:e2035057. 10.1001/jamanetworkopen.2020.35057 33410879 PMC7791354

[31] Centers for Disease Control and Prevention. Ending isolation and precautions for people with COVID-19: interim guidance [cited 2022 Aug 7]. https://www.cdc.gov/coronavirus/2019-ncov/hcp/duration-isolation.html

[32] Financial Markets Department, Bank of Japan. Foreign exchange rates [cited 2022 Aug 22]. https://www.boj.or.jp/en/statistics/market/forex/fxdaily/fxlist/fx220822.pdf

[33] Office for COVID-19 and Other Emerging Infectious Disease Control, Cabinet Secretariat, Government of Japan. Policies for COVID-19 emerging infectious disease control [in Japanese] [cited 2023 Feb 24]. https://corona.go.jp/surveillance

[34] Honda R, Murakami M, Hata A, Ihara M. Public health benefits and ethical aspects in the collection and open sharing of wastewater-based epidemic data on COVID-19. Data Sci J. 2021;20:27. 10.5334/dsj-2021-027

[35] Gable L, Ram N, Ram JL. Legal and ethical implications of wastewater monitoring of SARS-CoV-2 for COVID-19 surveillance. J Law Biosci. 2020;7:lsaa039. 10.1093/jlb/lsaa039 PMC7337755 32793373

[36] Ram N, Shuster W, Gable L, Ram JL. Ethical and legal wastewater surveillance. Science. 2023;379:652. 10.1126/science.adg7147 36795810

