# Supplementary material for: Economic Evaluation of Wastewater Surveillance Combined with Clinical COVID-19 Screening Tests, Japan
Source: Emerg Infect Dis. 2023 Aug;29(8):1608–17. doi: 10.3201/eid2908.221775 (PMC10370838; doi:10.3201/eid2908.221775)
Supplement: Appendix — Additional information on economic evaluation of wastewater surveillance combined with clinical COVID-19 screening tests, Japan. [file 22-1775-Techapp-s1.pdf]

Article DOI: https://doi.org/10.3201/eid2908.221775
EID cannot ensure accessibility for supplementary materials supplied by authors. Readers who have difficulty accessing supplementary content should contact the authors for assistance.

Economic Evaluation of Wastewater Surveillance Combined with Clinical COVID-19 Screening Tests, Japan

Appendix

Supplementary Methods

This supplemental section explains the details of the methods, mainly the parameters in this study's decision models. Appendix Figure 1 shows the possible scenarios for a hypothetical facility resident. Our base case analyses with a deterministic model assumed the point estimate for each parameter in Table 1.

Test Characteristics: Sensitivity and Specificity

Since PCR tests' sensitivity depends on the sampling procedures, our clinical PCR tests' sensitivity parameter was assumed to consist of two components. The first component is the sensitivity of clinical PCR tests using nasopharyngeal swabs as the standard, which was assumed to follow a triangular distribution with a mode of 89%, varying from 85.4%–91.8%, based on a systematic review (*1*).

The second component is a ratio that captures the sensitivity's uncertainties depending on sampling procedures, compared to the standard of nasopharyngeal swabs. Another systematic review estimated this ratio as follows: pooled nasal and throat swabs (97%), saliva swabs (85%),

nasal swabs (86%) and throat swabs (68%) (*2*). Based on these estimates, the second component ratio was assumed to follow a triangular distribution with a mode of 86% (i.e., a median of the four estimates above), ranging from 68%–97%.

We conducted a Monte Carlo simulation using these two components' distributions with 1,000 iterations, to obtain the mean (74%) with the 95% probabilistic confidence interval (PCI) of 64%–83%. Using these three estimates, our parameter on the sensitivity of clinical PCR tests (parameter name of sns_PCR in the decision tree) was assumed to follow a triangular distribution with a mode of 74%, varying from 64%–83%. The mode value of 74% was assumed to be a point estimate in a deterministic model.

Our study's antigen test is assumed to use rapid methods. Hence, results of an antigen test are assumed to be available within one hour after sampling. This rapid methods' sensitivity was estimated to be 72% by a systematic review study (*3*). This review study also estimated antigen tests' sensitivity to range from 40%–72%, depending on sampling procedures, e.g., nasopharyngeal samples or throat saliva samples (*3*). Using these estimates, our study assumed antigen tests' sensitivity to range from 40%–72% with a point estimate of 56%, which is the midpoint of this range.

Within one facility, the sensitivity of a primary screening with antigen tests was assumed to be lower than that of a secondary screening with clinical PCR tests. Thus, our study defined the antigen tests' sensitivity to consist of two components: the sensitivity of clinical PCR tests (described earlier) and the ratio of sensitivity of antigen test against clinical PCR tests that is <1. The latter ratio component (sns_Ag_ratio_PCR) was assumed to follow a triangular distribution with a mode of 76% (i.e., 56%/74% in which antigen tests' point estimate stated in the previous paragraph/mode of the clinical PCR tests' sensitivity defined earlier), varying from 54%

(40%/74%, in which antigen tests' minimum value/mode of the clinical PCR tests' sensitivity) to 97% (72%/74% = antigen tests' maximum value/mode of the clinical PCR tests' sensitivity).

As assumed in the previous paragraph, the sensitivity of a primary screening with antigen tests was lower than that of a secondary screening with clinical PCR tests. Therefore, when an infected case has a positive result in an antigen test, this case is highly likely to have a positive result in subsequent clinical PCR tests, e.g., 0.99 as a mode of these clinical PCR tests' sensitivity (sns_PCR_2nd). This parameter was assumed to range from 0.64 (i.e., the lower bound of the clinical PCR test defined above) to 0.999.

Our parameter on the specificity of antigen tests was to assume to follow a triangular distribution with a mode of 99%, varying from 96%–99.5%. The mode value of 99% was the median value of the eighteen studies examined in a systematic review (*3*). The minimum and the maximum values of these eighteen studies were 96%–99.5% (*3*).

The parameter on the specificity of PCR tests was assumed to follow a triangular distribution with a mode of 97.4%, varying from 96%–99.5%. The mode value of 97.4% was the weighted average of four sampling procedures among populations including asymptomatic subpopulations reported in a systematic review study (*2*). The minimum value of these four sampling procedures was 96% (*2*), which was used as a lower bound of the triangular distribution. The maximum value of 99.5% was assumed to be equal to that of antigen tests (*3*).

Cost of Tests

We defined two cost parameters to operate wastewater surveillance at a facility with a common unit of per day per facility: the laboratory cost and the labor cost to sample at a facility. Our parameter on the laboratory cost was assumed to follow a triangular distribution with a mode of $379, varying from $189–$758 (50%–200% of the mode). We used the exchange rate of

¥132 per $1 USD based on the time-series statistics data (annual average exchange rate) by the Bank of Japan in 2022 (*4*).

The mode value ($379 USD) was based on a price set by a laboratory company in Japan, which was similar to the cost of $300 USD reported by Safford and colleagues (*5*). We assumed that this price by a Japanese company included both a depreciation cost of the laboratory equipment (also called a fixed cost as an economics term) and a per-sample cost of consumables of reagents (also called a variable cost). The former and the latter costs were reported as $100,000 USD and $25 USD, respectively, by Kantor and associates (*6*), who did not report the total number of samples to completely depreciate the equipment cost. If this total number of samples is 295, the appropriate per-sample cost is estimated by summing a variable cost ($25) and a part of fixed costs ($339 = $100,000/295), i.e., $364 USD, which is equivalent to the point estimate of our cost parameter reported in the previous paragraph.

The labor cost to sample at a facility increased substantially if an additional operation at a public road outside a facility site, e.g., opening a manhole along a public road, was needed. This additional operation inflated the cost from $152 to $2,045 (*7*), which corresponded to a minimum value and a maximum value, respectively, in a triangle distribution assumed for this labor cost parameter. This distribution's mode value of $1,136 was close to a midpoint of this range.

The above two cost parameters were constant per facility per day regardless of the number of residents at a facility. Therefore, the cost of operating wastewater surveillance per facility resident declines, if "the number of residents at a facility" increases. Hence, the parameter on "the number of residents at a facility" is among cost related parameters. This parameter was assumed to follow a triangular distribution with a mode of 100, ranging from 50–200. The mode value was close to the maximum value among the four major types of long-term

care (LTC) facilities' average number of beds in Japan (*8*). The minimum value was close to the minimum value among these four types of LTC facilities (*8*). This distribution's maximum value of 200, assumed as 200% of the mode. Under a one-way sensitivity analysis of this parameter, the upper bound was 1,000, which was close to the smallest population size of a sampling area in the Tokyo Olympic and Paralympic Village in 2021 (*9*).

Our parameters for costs of clinical tests were defined based on the list of approved tests posted in the Japanese government website (*10*). Since the price information of these tests are limited, our parameters might be biased either upward or downward. In addition to the test cost, we assigned a labor cost to collect a test sample, which was calculated as the 30-minute time cost with the minimum wage ($7 USD per hour) in Japan, as explained in the footnote of Table 1 in the manuscript (*11*). Consequently, the parameter on antigen tests' cost was assumed to follow a triangular distribution with a mode of $16, varying from $10–$23. The parameter on clinical PCR tests' cost was assumed to follow a triangular distribution with a mode of $38, ranging from $20–$53.

Benefit of Confirming One Infected Case

Our study assumed the benefit of confirming one infected case by PCR tests under options 1 and 2, consisted of two components: the benefit of reducing hospitalization and mortality rates among the confirmed case and the benefit of preventing secondary infection. The former component is explained in this subsection.

Benefit of Reducing Hospitalization and Mortality Rates among Confirmed Cases

There is little literature that quantified the screening effectiveness in reducing hospitalization and mortality rates among a screened population. Thus, we assumed this effectiveness to follow the clinical efficacy of antiviral agents among non-hospitalized patients

with COVID-19. A systematic review based on a meta-analysis reported that this clinical efficacy was 77% in terms of lowering the risk of COVID-19–related hospitalization or death among cases within seven (or five) days after the onset of signs or symptoms (*12*). This review paper also estimated the efficacy for each of three types of antiviral agents, ranging from 33%–88% (*12*). In our study, these estimates were reduced by 30%, since 30% of infected individuals never develop symptoms (*13*). Consequently, our screening effectiveness was assumed to follow a triangular distribution with a mode of 0.54 (77% × [100%–30%]), ranging from 0.23 (33% × [100%–30%]) to 0.62 (88% × [100%–30%]) (Eff_early_Dx in Decision model). Our sensitivity analyses showed the robustness of our results regarding this parameter.

To assign benefit values for reducing hospitalization and mortality rates, we estimated the related monetary value for the three outcomes of isolation, hospitalization, and death among individuals confirmed as test positive. All individuals were assumed to be either isolated or hospitalized before a death. The hospitalization rate among test positives was assumed to follow a triangular distribution with a mode of 0.18, varying from 0.04–0.40 ("r_hosp_test_positive" in Decision model). Thus, under a base case analysis, 18% (the distribution mode was equal to a point estimate) and 82% (100% – 18%) among test-positives were hospitalized and isolated, respectively.

This distribution's mode value (0.18) was equal to the mean of the hospitalization rates among test-positives during a period from June 2, 2021–August 3, 2022 (*14*). The maximum value of 0.40 was equal to the average of the four hospitalization rates reported weekly from December 1–22, 2021 (i.e., as of December 1, 8, 15, and 22, 2021), when the epidemic level was very low and hence access to hospital care was guaranteed (*14*). The minimum value of 0.04 was equal to the average of the four rates reported weekly from June 1–22, 2022 (i.e., as of June 1, 8,

15, and 22, 2022), when the epidemic level was very high and hence access to hospital care was limited (*14*).

Isolation Related Cost

The isolation related cost included two cost items. The first cost item covers an isolation room with meals during the isolation period. The second item covers two additional PCR tests to end an isolation period. The first cost item was assumed to follow a triangular distribution with a mode of $758, ranging from $379–$1,515 (C_isolation in Decision model), based on the specific fee schedule set by the Japanese government (*15*).

Hospitalization Cost

Among hospitalized cases, three severity levels were assumed to follow the Japanese government fee-for-service (FFS)–based payment rates (*16*). The proportion of most severe cases was estimated to have a mean of 10%, varying from 5%–19%, based on the weekly variations during a period from June 2, 2021–August 3, 2022 (*14*). Using these estimates, the proportion of severe cases among hospitalized cases was assumed to follow a triangular distribution with a mode of 0.1, varying from 0.05–0.19 ("r_icu_hosp" in Decision model).

Among non-severe cases, the percentage of the light cases were estimated to be 64% and moderate cases were estimated to be 36% based on the publicly available data in Japan (*16*). Thus, the proportions of three severity levels were estimated (Appendix Table 1). For instance, when the proportion of severe cases was 10% (at its mode), the remaining proportions of light were estimated to be 58% and moderate cases were estimated to be 32%.

To estimate a total cost for a hospitalized case, a supplemental payment rate should be added to the FFS–based payment rate (Appendix Table 2). There is a marked difference between these two payment rates. That is, the FFS–based payment rate was applied only for an actual

admission. On the other hand, a supplemental payment rate is paid for a hospital that secured a hospital bed for "potential" COVID-19 admissions, even if this bed is not occupied (*17*). Since our analysis adopted the societal viewpoint, the payments made for unoccupied beds should be assigned for admitted patients. For instance, if the bed occupancy rate is 50%, the average payment rates per an actual admission should be inflated twice (i.e., 100% ÷ 50%). The observed occupancy rate was variable, e.g., <10% for a certain period, which seemed difficult to assign a reasonable distribution. To make the most conservative estimates, the values in the column of "Supplemental payment rate per day per case" in Appendix Table 2 assumed the bed occupancy rate of 100%. Our conservative estimates are likely to underestimate the total hospitalization costs.

For each of three columns in Appendix Table 1, the weighted average of the total hospitalization cost was estimated. For instance, when the proportion of severe cases was 10%, the weighted total hospitalization cost was around \$19,384 (\$10,322 × 58% + \$17,731 × 32% + \$77,229 × 10%; weighting severity-specific costs listed in the far-right column of Appendix Table 2). Likewise, when the proportions of severe cases were 5% the total hospitalization cost estimates were ≈\$16,212 and at 19% were ≈\$25,227. Using these three estimates, a linear association was assumed between "the proportion of severe cases (r_icu_hosp)" and "total hospitalization cost (C_hosp_all)," mathematically expressed below:

$$(C\_hosp\_all) = \$16{,}212 + \$64{,}394 \times (r\_icu\_hosp - 0.05)$$

Death Related Value

We assigned monetary values for deaths due to COVID-19 by applying the monetary value (\$37,879) for a quality adjusted life year (QALY) saved or lost under the cost-effectiveness analysis set by Japan's Ministry of Health, Labor and Welfare (*18*). To estimate

QALYs lost due to COVID-19, we first calculated life years lost based on "age at death" (*19*) Appendix Tables 3, and "life expectancy among a certain age and gender group," (*20*) Appendix Tables 4, where age groups were classified into 10 groups, e.g., "<10 years old," "10–19," and "≥90."

Since we used sex-specific life expectancies from Appendix Table 4, we disregarded the data in the third and far right columns in Appendix Table 3 where sex was not disclosed. For simplicity, we assumed that the average age of each age category in Appendix Table 3 is a mid-point, e.g., age 15 for the age group of "10–19." As an exception, the average for the oldest age group of "≥90" in Appendix Table 3 was assumed to be 90, since the life expectancy value is not available for age ≥ 90 years in the sources of Appendix Table 4 (*20*).

The life years lost among the group of "male and age at 10s (average age of 15)" was equal to "66.89 (2nd row, 1st column in Appendix Table 4)." Applying this calculation for all sex-age groups in Appendix Table 3 (left columns of data up to December 27, 2021, when the "5th wave" ended in Japan), the average lost life years (weighted by the age-sex distribution) was estimated to be 11.7 years (not presented in tables). Using other death data in this table (left columns of data up to August 1, 2022, most recent data analyzed), this estimate was 11.1 years. Using these two estimates, the parameter on life years saved (V_life_yrs_saved in Decision model) was assumed to follow a triangular distribution, ranging from 11.1–11.7, with a mode of 11.4 (the average of these two estimates).

To convert "life years lost" to "QALYs lost," we applied the ratios among the Dutch population (*21*), due to the absence of relevant data in Japan. Wouterse and associates estimated that the sex-specific ratios were 0.71 (men) and 0.64 (women) (*21*). Using these two estimates, the parameter on the ratio to convert "life years lost" to "QALYs lost" (r_QALY_adj in Decision

model) was assumed to follow a triangular range from 0.64–0.71, with a mode of 0.68. This mode value was the weighted average of these two estimates, where weights were the sex proportions among cumulative COVID-19 deaths (up to August 1, 2022) in Japan, i.e., 0.584 (men) and 0.416 (women) (*19*).

Since our interest is to measure the benefit of confirming test positive cases by a screening, we estimated "the mortality rates among test positives" for each of the general population and the subpopulation ≥80 years of age. The latter age group was selected because average age of LTC facility residents was around 86 in Japan (*22*). The COVID-19 mortality rates could vary due to a population vaccination rate and viral variants unique to each epidemic wave. Thus, we estimated the mortality rates during the three periods: up to the 6th wave (September 8, 2020–May 31, 2022), up to the 5th wave (September 8, 2020–January 4, 2022), and during the 6th wave (January 4, 2022–May 31, 2022), excluding a period of the ongoing 7th wave as of August 25, 2022 (*23*) (Appendix Table 5).

Additionally, since the average hospital length of stay among severe cases was 21 days (*16*), the data period for deaths (numerator) was extended for 1–4 weeks (Appendix Table 5). Of note, age-specific mortality rates were available only after September 8, 2020 (*23*). Using the estimates in this table, the model parameter on the mortality rate among test positives (r_mortality_test_positive in Decision model) was assumed to follow a triangular distribution with a mode of 0.0035, ranging from 0.0018–0.0104 among the general population. The parameter concerning the ratio of mortality rate among persons ≥80 years of age, compared to the general population (V_mr_ratio) was assumed to follow a triangular distribution with a mode of 19, varying from 15–22, based on the estimates in this table (*23*).

Based on the parameters defined thus far, we estimated the monetary value of one confirmed case regarding the hospitalization and the mortality rates under a base case analysis among the general population, as mathematically expressed below.

Value of hospitalization for one confirmed case = Hospitalization rate × Hospitalization cost per admission, i.e., 18% × \$19,384 per admission = \$3,489

Value of mortality for one confirmed case = Mortality rate × Life years lost × Conversion to QALY × Value per QALY, i.e., 0.0035 × 11.4 × 0.68 × \$37,879 = \$1,028

Among the LTC residents, these values above will be inflated by 19, which assumes that the ratio of age-specific hospitalization rates was equal to that of mortality rates derived from the estimates in Appendix Table 5 (*23*). This is because there was no literature concerning the ratio of age-specific hospitalization rates in Japan.

Consequently, when the screening effectiveness in reducing hospitalization and mortality rates because of an earlier diagnosis (Eff_early_Dx) is assumed as 0.54 under our base case analysis, its benefit is expressed below.

Benefit of reducing hospitalization and mortality rate for a confirmed case = 0.54 × (values of hospitalization + value of mortality) × Ratio of mortality/hospitalization among persons $\geq$80 years of age = 0.54 × (\$3,489 + \$1,028) × 19 = \$46,343

Benefit of Preventing Secondary Infection

As explained earlier, our study assumed the benefit of confirming one infected case by PCR tests under both option 1 and option 2, consisted of two components: the benefit of reducing hospitalization and mortality rates among the confirmed cases and the benefit of preventing secondary infection. The latter component is explained in this subsection.

Appendix Figure 2 shows our calculation method to quantify the number of secondary infected cases prevented by a confirmatory PCR test. Two key parameters are the reproduction number (R_e in this figure) on the figure's y-axis and the infectious period (e.g., 10 days (*13*) assumed in this figure) on the x-axis. Also, the number of preventable secondary infected cases depends on "screening timing during an infectious period (that cannot be observed)" and "time lag between screening timing and isolation timing."

The best-case scenario was indicated by a point in Appendix Figure 2, where the PCR sample was collected at the moment when an infectious period starts (i.e., 0 on the x-axis). Under this best-case scenario, the PCR result is available immediately and hence the infected case is isolated without producing any secondary infected cases. Therefore, the number of prevented secondary infections is R_e (i.e., the best-case scenario point's y-axis value).

Another extreme scenario is the worst-case scenario, also noted by a point in Appendix Figure 2. Under this worst-case scenario, the PCR sample was collected at the moment when the infectious period ends on day 10 (i.e., the worst-case scenario point's x-axis value). Due to such a delayed sampling timing, the number of preventable secondary infections is zero (i.e., the worst-case scenario point's y-axis value) even if the PCR test is available immediately. This is because the confirmed case has already produced secondary infected cases (with the magnitude of R_e) and hence would not produce any more secondary infected cases.

Our decision models assumed more realistic "second-best case scenario" and "second-worst case scenario." This is mainly because we assumed that time lag between screening timing and isolation timing was 0.5 day. Under this more realistic "second-best case scenario," the PCR sample collection timing is the same as the best-case scenario explained above, at the moment when an infectious period starts (i.e., 0 on the x-axis). However, during the time lag before an

isolation, the infected case has already produced secondary infection with the magnitude of $R_e \times (0.5 \div 10)$. Still, the PCR test prevented secondary infection with the magnitude of $R_e \times (10 - 0.5) \div 10$, which is the second-best case scenario point's y-axis value. For simplicity, these calculations assumed that the first generation confirmed case discharges a constant number of viruses during the infectious period.

Under the second-worst-case scenario, the number of preventable secondary infections is 0 (i.e., the second-worst-case scenario point's y-axis value), even though the PCR sampling timing is 0.5 day earlier than the worst-case scenario. This is because when the confirmed case is isolated, this first generation confirmed case stops producing secondary infections.

Accounting for all possible scenarios with any prevented secondary infected cases, the sum of the prevented secondary infected cases is equal to the triangular area (with darker shadow in black and white/red-and-blue stripes) defined by the second-best-case scenario point, the second-worst-case scenario point and the origin point in Appendix Figure 2, i.e., $0.5 \times (Re \times [(10 - 0.5) \div 10]) \times 9.5$. Thus, the average number of prevented secondary infected cases per day, during the 10-day infectious period, is estimated as $0.5 \times (Re \times [(10 - 0.5) \div 10]) \times 9.5 \times 1/10$.

As explained above, the average number of prevented secondary infected cases is affected by an infectious period that varies among test positives. The US Centers for Disease Control and Prevention (CDC) guideline recommended different isolation periods depending on the three severity levels among test positives (*24*), which are different from the three severity levels among hospitalized in Japan described earlier. This CDC guideline recommended isolation periods of 5, 10, and 20 days, after symptom onset. Since the infectious period is reported as 2 days before symptom onset (*24*), the infectious periods were assumed to be 7, 12, and 22 days in our decision models.

The weighted average of the infectious periods was estimated by assigning the probability for each of three infectious periods. Regarding the mild cases with a 7-day infectious period, its probability was assumed to be equal to that of isolation among test positives (e.g., 82% under a base-case analysis). For severe cases with a 12-day infectious period, its probability was assumed to be equal to that of hospitalization among test positives, excluding the severe level among the hospitalized (e.g., 18% × 90% under a base-case analysis). Concerning immunocompromised cases with a 22-day infectious period, its probability was assumed to be equal to that of hospitalized with the severe level among test positives (e.g., 18% × 10% under a base-case analysis). Using these assumptions, the weighted average of the infectious periods was estimated to be 8.08 days under the base-case analysis, which was slightly shorter than an estimate of 10 days by Johansson and colleagues (*13*).

Consequently, the average number of prevented secondary infected cases during a certain infectious period (T) is estimated as $0.5 \times (Re \times [(T - 0.5)/T]) \times (T - 0.5) \times (1/T)$. Under the base case analysis (when R_e is 1.3 and T is 8.08), the estimate of the prevented secondary infection was 0.57. Since we assume that the secondary infected case has the same values of hospitalization and mortality rates as a first generation confirmed case stated earlier, the benefit of preventing secondary infection was estimated with the equation below.

Benefit of preventing secondary infection = 0.57 × 0.54 × (value of hospitalization + value of mortality) × ratio of hospitalization/mortality rates among persons ≥80 years of age; thus, 0.57 × 0.54 × ($3,489 + $1,028) × 19 = $26,415 under the base-case analysis.

Finally, the total benefit of finding one confirmed case by a screening was estimated with the equation below.

Total benefit of finding one confirmed infected case by a screening (Bnft_per_case in Decision tree) = [Benefit of reducing hospitalization and mortality rate for a first generation confirmed case]

+ [Benefit of preventing secondary infection] = [$46,343] + [$26,415] = $72,758 under the base-case analysis

Loss of Missing an Infected Case (Test False-Negative Case)

Our study also modeled the loss due to missing a first-generation infected case, i.e., a test false negative case, besides the benefit of finding an infected case explained above. Our model estimated how many second-generation infected cases were produced by a first-generation infected case that was not detected by our screening test for each day from day 1 to day 4. In other words, in our decision model's terminal node, the benefit of finding one infected case was reduced by the loss due to an infected case that already produced a second-generation infected case.

Appendix Figure 3, panel A assumed a 10-day infectious period, following the assumptions in Appendix Figure 2 above. Under Appendix Figure 3, panel B, an infectious period is expressed as "t," which was assigned a triangular distribution in our probabilistic analysis explained earlier.

Appendix Figure 3, panel A indicates that a first-generation infected case produces second-generation infected cases with the magnitude of R_e (i.e., triangular area = $(1/2) \times (R_e/5) \times 10$) during the 10 infectious period. This magnitude is assumed to decline from day 1 to day 10, illustrated by a downslope. Since we do not know when an infected case became infectious, we assumed that the infected case can be from day 1 to day 10, with a 10% probability for each day.

Therefore, on day 1, if a first-generation infected case is missed by the false-negative result of a screening test, on the average, this "missed" first-generation infected case produces second-generation infected cases with the magnitude of [(a1+a2+a3+ ⋯+a10)/10] (Appendix Figure 3, panel A).

Namely, the number of newly produced second-generation infected cases is (1/10) × (1/2) × (R_e/5) × (10) = (R_e/10)

This magnitude is equivalent to [(a1+a2+a3+ ⋯+at)/t] in Appendix Figure 3, panel A, in which (1/t) × (1/2) × (R_e/(t/2)) × (t) = (Re/t), expressed as (Inf_2nd_D1) in Appendix Figure 1.

On day 2, all first-generation infected cases were assumed to shift rightward in Appendix Figure 3, since these infected cases aged by 1 day. As a result, in Appendix Figure 3, the triangular area on day 2 became smaller in magnitude than that on day 1. The difference in area between day 1 and day 2 was equivalent to the far left trapezoid area of a1 in Appendix Figure 3.

Thus, on day 2, if a first-generation infected case is missed by the false-negative result of a screening test, on the average, this "missed" first-generation infected case produces second-generation infected cases with the magnitude of [(a2+a3+a4+⋯+a10)/9] in Appendix Figure 3, panel A.

Namely, the number of newly produced second-generation infected cases = (1/9) × (1/2) × [(R_e/5) × (9/10)] × 9 = (R_e/10) × (9/10). This magnitude is equivalent to [(a2+a3+ ⋯+at)/(t – 1)] in Appendix Figure 3, panel B, in which 1/(t – 1) × (1/2) × [(R_e/(t/2)) × (t – 1) × (1/t)] × (t – 1) = (R_e/t) × (t – 1) × (1/t), expressed as (Inf_2nd_D2) in Appendix Figure 1.

Likewise, on day 3, if a first-generation infected case is missed by the false-negative result of a screening test, on the average, this "missed" first-generation infected case produces

second-generation infected cases with the magnitude of [(a3+a4+⋯+a10)/8] in Appendix Figure 3, panel A.

Namely, the number of newly produced second-generation infected cases is (1/8) × (1/2) × [(R_e/5) × (8/10)] × 8 = (R_e/10) × (8/10). This magnitude is equivalent to [((a3+a4+ ⋯+at)/(t – 2)] in Appendix Figure 3, panel B, in which 1/(t – 2) × (1/2) × [(R_e/(t/2)) × (t – 2) × (1/t)] × (t – 2) = (R_e/t) × (t – 2) × (1/t), expressed as (Inf_2nd_D3) in Appendix Figure 1.

Similarly, on day 4, if a first-generation infected case is missed by the false-negative result of a screening test, on the average, this "missed" first-generation infected case produces second-generation infected cases with the magnitude of [(a4+a5+⋯+a10)/7] in Appendix Figure 3, panel A. Namely, the number of newly produced second-generation infected cases is (1/7) × (1/2) × [(R_e/5) × (7/10)] × 7 = (R_e/10) × (7/10).

This magnitude is equivalent to [((a4+a5+ ⋯+at)/(t-3)) in Appendix Figure 3, panel B, in which (1/(t – 3)) × (1/2) × [(R_e/(t – 3)) × (t – 3) × (1/t)] × (t – 3) = (R_e/t) × (t – 3) × (1/t), expressed as (Inf_2nd_D4) in Appendix Figure 1.

Additionally, our study assumed that the value of missing one infected case (Loss_per_case) is equal to that of finding one infected case (Bnft_per_case) under each screening option, under the base case analysis. Our one-way sensitivity analysis assumed the ratio of Loss/Benefit to range from 0 to 2. The results of this one-way sensitivity analysis was robust (Appendix Table 28).

"Total benefit of finding one confirmed case by a screening (Bnft_per_case in Decision tree)]" was defined earlier. In our modeling, on day 2, even when one infected case was detected by a screening test, the benefit was discounted by allowing this case to produce secondary infected cases on day 1, expressed as below, when the infectious period is t:

$Bnft\_per\_case \times (1 - (R_e/t))$

Under the base case analysis, this equation is expressed as below:

$Bnft\_per\_case - Loss\_per\_case \times Inf\_2nd\_D1$

where the term of (R_e/t) is explained with Appendix Figure 3, panel B, and defined as Inf_2nd_D1 earlier.

Likewise, on day 3, even when one infected case was detected by a screening test, the benefit was discounted by allowing this case to produce secondary infected cases on day 1 and day 2, expressed as below, when the infectious period is t:

$Bnft\_per\_case \times (1 - (R_e/t) - (R_e/t) \times (t - 1) \times (1/t))$

Under the base case analysis, this equation is expressed as below:

$Bnft\_per\_case - Loss\_per\_case \times (Inf\_2nd\_D1 + Inf\_2nd\_D2)$

where the term of $(R_e/t) \times (t - 1) \times (1/t)$ is explained with Appendix Figure 3, panel B, and defined as Inf_2nd_D2 earlier.

Similarly, on day 4, even when one infected case was detected by a screening test, the benefit was discounted by allowing this case to produce secondary infected cases on day 1, day 2, and day 3, expressed as below, when the infectious period is t:

$Bnft\_per\_case \times (1 - (R_e/t) - (R_e/t) \times (t - 1) \times (1/t) - (R_e/t) \times (t - 2) \times (1/t))$

Under the base case analysis, this equation is expressed as below:

$Bnft\_per\_case - Loss\_per\_case \times (Inf\_2nd\_D1 + Inf\_2nd\_D2 + Inf\_2nd\_D3)$

where the term of $(R_e/t) \times (t - 2) \times (1/t)$ is explained with Appendix Figure 3, panel B, and defined as Inf_2nd_D3 earlier.

On day 4, when one infected case was still not detected by a screening test, the loss of missing this infected case is expressed as below, when the infectious period is t:

$$-Bnft\_per\_case \times ((R_e/t) + (R_e/t) \times (t - 1) \times (1/t) + (R_e/t) \times (t - 2) \times (1/t) + (R_e/t) \times (t - 3) \times (1/t))$$

Under the base case analysis, this equation is expressed as below:

$$- Loss\_per\_case \times (Inf\_2nd\_D1 + Inf\_2nd\_D2 + Inf\_2nd\_D3 + Inf\_2nd\_D4)$$

where the term of $(R_e/t) \times (t - 3) \times (1/t)$ is explained with Appendix Figure 3, panel B, and defined as Inf_2nd_D4 earlier.

Relationship between Prevalence and Incidence

Our decision model assumed the prevalence as a function of the incidence. To define a relatively simplified relationship between the COVID-19 disease prevalence and incidence, we assumed the hypothetical scenario among N residents in the area around the facility illustrated in Appendix Figure 4. In this figure, the top line shows a date from the first case being infected. This infected case was assumed to be infectious from day 3 to day 13, i.e., for a 10-day period (*13*). Only during this 10-day infectious period, could an infected case be detected by antigen tests and PCR tests, i.e., counted as a part of incidence (presented as the bottom line in this figure) and prevalence (2nd line from the bottom in this figure). Therefore, on day 3, both the prevalence and the incidence were one [per N residents].

The first infected case was also called the 1st generation in this figure. During the 10-day infectious period, the first infected case transmits viruses to produce 2nd generation infected cases at the magnitude of R, which is a reproduction number. For simplicity, the 2nd generation infected cases were produced at the midpoint of the 10-day infection period, i.e., day 8. Thus, on

day 8 the incidence was R [per N residents] and the prevalence was "R (2nd generation) plus one (1st generation is still infectious)."

Similarly, the 3rd generation infected cases were produced at the midpoint of the 10-day infection period, i.e., day 13. Since each of R infected cases among the 2nd generation produces newly infected cases by R, the 3rd generation infected cases were "R-squared (R^2)" in number. Hence, on day 13, the incidence was "R^2" [per N residents] and the prevalence was "R^2 (3rd generation) plus R (2nd generation) plus one (the final infectious day of 1st generation)."

Likewise, on day 18, the incidence was "R^3" [per N residents] and the prevalence was "R^3 (4th generation) plus R^2 (3rd generation) plus R (2nd generation)." On day 23, the incidence was "R^4" [per N residents] and the prevalence was "R^4 plus R^3 plus R^2."

After day 13, a proxy for the relationship between prevalence and incidence is mathematically expressed as below:

A proxy of "Prevalence/Incidence" = $(1 + R + R^2)/ (R^2)$

This proxy ratio was 2.36 in our base case analysis, where the reproduction number (R in this figure) is at the point estimate of 1.3. When the reproduction number ranged from 0.9 to 2, this ratio declined from 3.35 to 1.75. This point estimate and this range of the reproduction number followed Neilan et al (*25*).

In the decision tree, the disease prevalence (pvl_fx_Re) was defined as a multiplication of the disease incidence and a proxy of "Prevalence/Incidence" expressed above.

References

1. Marando M, Tamburello A, Gianella P, Taylor R, Bernasconi E, Fusi-Schmidhauser T. Diagnostic sensitivity of RT-PCR assays on nasopharyngeal specimens for detection of SARS-CoV-2

infection: a systematic review and meta-analysis. Caspian J Intern Med. 2022;13(Suppl 3):139–47. PubMed

2. Tsang NNY, So HC, Ng KY, Cowling BJ, Leung GM, Ip DKM. Diagnostic performance of different sampling approaches for SARS-CoV-2 RT-PCR testing: a systematic review and meta-analysis. Lancet Infect Dis. 2021;21:1233–45. PubMed https://doi.org/10.1016/S1473-3099(21)00146-8

3. Tapari A, Braliou GG, Papaefthimiou M, Mavriki H, Kontou PI, Nikolopoulos GK, et al. Performance of antigen detection tests for SARS-CoV-2: a systematic review and meta-analysis. Diagnostics (Basel). 2022;12:1388. PubMed https://doi.org/10.3390/diagnostics12061388

4. Bank of Japan. Time-series data search [in Japanese] [cited 2023 Feb 14]. https://www.stat-search.boj.or.jp/ssi/cgi-bin/famecgi2?cgi=$nme_a000&lstSelection=FM08

5. Safford HR, Shapiro K, Bischel HN. Opinion: Wastewater analysis can be a powerful public health tool-if it's done sensibly. Proc Natl Acad Sci U S A. 2022;119:e2119600119. PubMed https://doi.org/10.1073/pnas.2119600119

6. Kantor RS, Greenwald HD, Kennedy LC, Hinkle A, Harris-Lovett S, Metzger M, et al. Operationalizing a routine wastewater monitoring laboratory for SARS-CoV-2. PLOS Water. 2022;1:e0000007. https://doi.org/10.1371/journal.pwat.0000007

7. Ministry of Land Infrastructure, Transport and Tourism. Engineer unit price for a design outsourcing for fiscal year 2021 [in Japanese] [cited 2022 Aug 23]. https://www.mlit.go.jp/tec/content/001387446.pdf

8. Ministry of Health Labour and Welfare. Overview of survey of institutions and establishments for long-term care for FY 2020 [in Japanese]. https://www.mhlw.go.jp/toukei/saikin/hw/kaigo/service20/dl/kekka-gaiyou_1.pdf

9. Kitajima M, Murakami M, Kadoya SS, Ando H, Kuroita T, Katayama H, et al. Association of SARS-CoV-2 Load in Wastewater With Reported COVID-19 Cases in the Tokyo 2020 Olympic and Paralympic Village From July to September 2021. JAMA Netw Open. 2022;5:e2226822. PubMed https://doi.org/10.1001/jamanetworkopen.2022.26822

10. Ministry of Health Labour and Welfare. Approval information for in-vitro diagnostic reagents (test kits) for COVID-19 [in Japanese] [cited 2022 Aug 22]. https://www.mhlw.go.jp/stf/newpage_11331.html

11. Ministry of Health Labour and Welfare. Current status of minimum wage amendments by regions for FY 2021 [in Japanese] [cited 2022 Aug 4]. https://www.mhlw.go.jp/stf/seisakunitsuite/bunya/koyou_roudou/roudoukijun/minimumichiran

12. Lai CC, Wang YH, Chen KH, Chen CH, Wang CY. The clinical efficacy and safety of anti-viral agents for non-hospitalized patients with COVID-19: a systematic review and network meta-analysis of randomized controlled trials. Viruses. 2022;14:1706. PubMed https://doi.org/10.3390/v14081706

13. Johansson MA, Quandelacy TM, Kada S, Prasad PV, Steele M, Brooks JT, et al. SARS-CoV-2 transmission from people without COVID-19 symptoms. JAMA Netw Open. 2021;4:e2035057. PubMed https://doi.org/10.1001/jamanetworkopen.2020.35057

14. Ministry of Health Labour and Welfare. Ministry of Health, Labour and Welfare (MHLW). Survey on medical treatment and number of inpatient beds [in Japanese] [cited 2021 Sep 14]. https://www.mhlw.go.jp/stf/seisakunitsuite/newpage_00023.html

15. Ministry of Health Labour and Welfare. Handling of the emergency comprehensive support program (for medical care) for COVID-19 in FY 2022 [in Japanese] [cited 2022 Aug 31]. https://www.mhlw.go.jp/content/000968054.pdf

16. Global Health Consulting. The unit price of a COVID-19 patient - 54,000 yen for mild cases, 80,000 yen for moderate cases, and 142,000 yen for severe cases - is not worth the investment cost, according to the president of Japan Municipal Hospital Association (JMHA) [inn Japanese] [cited 2021 Nov 24]. https://gemmed.ghc-j.com/?p=38100

17. Ministry of Health Labour and Welfare. Amendments for the rules on "major medical institutions for COVID-19 and cooperative medical institutions accepting suspected COVID-19 patients" [in Japanese] [cited 2022 Aug 31]. https://www.mhlw.go.jp/content/000764832.pdf

18. Central Social Insurance Medical Council, Ministry of Health, Labour and Welfare (MHLW). Examination of scientific matters for cost-effectiveness evaluation (part 4) [in Japanese] [cited 2022 Aug 31]. https://www.mhlw.go.jp/content/12404000/000380564.pdf

19. National Institute of Population and Social Security Research (IPSS). Data on COVID-19 [cited 2022 Aug 31]. www.ipss.go.jp/projects/j/Choju/covid19/index-en.asp

20. Ministry of Health Labour and Welfare. Abridged life tables for Japan 2020 [cited 2022 Aug 25]. https://www.mhlw.go.jp/english/database/db-hw/lifetb20/dl/lifetb20-06.pdf

21. Wouterse B, Ram F, van Baal P. Quality-adjusted life-years lost due to COVID-19 mortality: methods and application for The Netherlands. Value Health. 2022;25:731–5. PubMed https://doi.org/10.1016/j.jval.2021.12.008

22. e-Stat. Survey of institutions and establishments for long-term care – result detail for FY 2019 [in Japanese] [cited 2022 Jun 16]. https://www.e-stat.go.jp/stat-search/files?&stat_infid=000032069585

23. Ministry of Health Labour and Welfare. Visualizing the data: information on COVID-19 infections [in Japanese] [cited 2022 Jul 26]. https://covid19.mhlw.go.jp/extensions/public/index.html

24. US Centers for Disease Control and Prevention (CDC). Ending isolation and precautions for people with COVID-19: interim guidance [cited 2022 Aug 7]. https://www.cdc.gov/coronavirus/2019-ncov/hcp/duration-isolation.html

25. Neilan AM, Losina E, Bangs AC, Flanagan C, Panella C, Eskibozkurt GE, et al. Clinical impact, costs, and cost-effectiveness of expanded severe acute respiratory syndrome coronavirus 2 testing in Massachusetts. Clin Infect Dis. 2021;73:e2908–17. PubMed https://doi.org/10.1093/cid/ciaa1418

Appendix Table 1. Percentage of three severity levels among COVID-19 hospitalized cases in Japan*

Severity level	Mode (point estimate) of severe cases	Minimum severe cases	Maximum severe cases
Light	58%	61%	52%
Moderate	32%	34%	29%

*The mode (point estimate) of severe cases was 10%; minimum was 5% and maximum was 19%.

Appendix Table 2. Total payment rates per hospitalized case based on COVID-19 severity, Japan*

Severity level	Average length of stay, d†	FFS based payment rate per day per case†	Supplemental payment rate per day per case‡	Total hospitalization cost per case
Light	10.9	$409	$538	$10,322
Moderate	15.5	$606	$538	$17,731
Severe	22	$1,076	$2,435	$77,229

*Cost reported in USD; the exchange rate of ¥132 per $1 USD was used as the annual average in 2022 based on the report by the Bank of Japan (*4*). FFS, fee for service.

†Average length of stay and FFS from Global Health Consulting (*16*).

‡Supplemental payment rate from Ministry of Health, Labour and Welfare (*17*).

Appendix Table 3. Age and sex distribution among deaths due to COVID-19, Japan*

	Cumulative deaths up to 2021 Dec 27			Cumulative deaths up to 2022 Aug 1		
Age	M	F	Not disclosed	M	F	Not disclosed
<10	0	0	0	4	4	0
10–19	2	1	0	6	2	1
20–29	15	5	2	26	8	2
30–39	54	19	2	74	30	5
40–49	188	53	7	254	73	9
50–59	569	129	15	725	182	30
60–69	988	292	34	1,369	406	55
70–79	2,333	1,056	74	3,596	1,535	134
80–89	3,134	2,519	252	5,177	3,836	209
≥90	1,096	1,814	33	2,129	3,459	164

*Data collected from National Institute of Population and Social Security Research (*19*).

Appendix Table 4. Life expectancies by sex at specific ages in Japan in 2020*

	Life expectancy, y	
Current age, y	M	F
5	76.83	82.93
15	66.89	72.98
25	57.12	63.12
35	47.40	53.28
45	37.80	43.56
55	28.58	34.09
65	20.05	24.91
75	12.63	16.25
85	6.67	8.76
90	4.59	5.92

*Data collected from Ministry of Health, Labour and Welfare (*20*).

Appendix Table 5. Mortality rates among persons testing SARS-CoV-2 positive during different waves, Japan (*23*)

	Up to the 6th wave†		Up to the 5th wave‡		During the 6th wave§	
Time lag*	Date range	Mortality rate, %	Date range	Mortality rate, %	Date range	Mortality rate, %
Persons >80 y						
0	2020 Sep 8–2022 May 31	0.35	2020 Sep 8–2022 Jan 4	1.03	4 Jan–31 May 2022	0.18
1 week	2020 Sep 8–2022 Jun 7	0.35	2020 Sep 8–2022 Jan 11	1.03	4 Jan–7 Jun 2022	0.18
2 weeks	2020 Sep 8–2022 Jun 14	0.35	2020 Sep 8–2022 Jan 18	1.03	4 Jan–14 Jun 2022	0.18
3 weeks	2020 Sep 8–2022 Jun 21	0.35	2020 Sep 8–2022 Jan 25	1.04	4 Jan–21 Jun 2022	0.19
4 weeks	2020 Sep 8–2022 Jun 28	0.35	2020 Sep 8–2022 Feb 1	1.05	4 Jan–28 Jun 2022	0.19
Persons ≥80 y						
0	2020 Sep 8–2022 May 31	6.70	2020 Sep 8–2022 Jan 4	15.34	4 Jan–31 May 2022	4.01
1 week	2020 Sep 8–2022 Jun 7	6.74	2020 Sep 8–2022 Jan 11	15.34	4 Jan–7 Jun 2022	4.06
2 weeks	2020 Sep 8–2022 Jun 14	6.77	2020 Sep 8–2022 Jan 18	15.36	4 Jan–14 Jun 2022	4.10
3 weeks	2020 Sep 8–2022 Jun 21	6.81	2020 Sep 8–2022 Jan 25	15.45	4 Jan–21 Jun 2022	4.16
4 weeks	2020 Sep 8–2022 Jun 28	6.84	2020 Sep 8–2022 Feb 1	15.71	4 Jan–28 Jun 2022	4.19

*Time lag is the period between positive tests (denominator) and death (numerator); date range indicates range during which deaths were included.

†September 8, 2020–May 31, 2022.

‡September 8, 2020–January 4, 2022.

§January 4–May 31, 2022.

Appendix Table 6. One-way sensitivity analysis for sensitivity of wastewater surveillance used in an economic evaluation of wastewater surveillance combined with clinical COVID-19 screening tests, Japan*

	Option 1†			Option 2†			Relative value of option 2		
Sensitivity	Cost	Benefit	ROI‡	Cost	Benefit	ROI‡	Inc. cost§	Inc. benefit¶	Rel. ROI#
0.46	$67.05	$14.09	0.21	$53.61	$6.69	0.12	–$13.44	–$7.40	2
0.47	$67.05	$14.09	0.21	$53.61	$7.05	0.13	–$13.44	–$7.04	2
0.48	$67.05	$14.09	0.21	$53.61	$7.40	0.14	–$13.44	–$6.69	2
0.49	$67.05	$14.09	0.21	$53.61	$7.74	0.14	–$13.44	–$6.35	2
0.5	$67.05	$14.09	0.21	$53.61	$8.07	0.15	–$13.44	–$6.02	2
0.51	$67.05	$14.09	0.21	$53.61	$8.39	0.16	–$13.43	–$5.70	2
0.52	$67.05	$14.09	0.21	$53.61	$8.70	0.16	–$13.43	–$5.39	2
0.53	$67.05	$14.09	0.21	$53.61	$8.99	0.17	–$13.43	–$5.10	3
0.54	$67.05	$14.09	0.21	$53.61	$9.28	0.17	–$13.43	–$4.81	3
0.55	$67.05	$14.09	0.21	$53.61	$9.55	0.18	–$13.43	–$4.54	3
0.56	$67.05	$14.09	0.21	$53.61	$9.81	0.18	–$13.43	–$4.28	3
0.57	$67.05	$14.09	0.21	$53.61	$10.07	0.19	–$13.43	–$4.02	3
0.58	$67.05	$14.09	0.21	$53.61	$10.31	0.19	–$13.43	–$3.78	4
0.59	$67.05	$14.09	0.21	$53.61	$10.55	0.20	–$13.43	–$3.54	4
0.6	$67.05	$14.09	0.21	$53.61	$10.77	0.20	–$13.43	–$3.32	4
0.61	$67.05	$14.09	0.21	$53.61	$10.99	0.20	–$13.43	–$3.10	4
0.62	$67.05	$14.09	0.21	$53.61	$11.20	0.21	–$13.43	–$2.89	5
0.63	$67.05	$14.09	0.21	$53.61	$11.39	0.21	–$13.43	–$2.70	5
0.64	$67.05	$14.09	0.21	$53.61	$11.58	0.22	–$13.43	–$2.51	5
0.65	$67.05	$14.09	0.21	$53.61	$11.77	0.22	–$13.43	–$2.32	6
0.66	$67.05	$14.09	0.21	$53.61	$11.94	0.22	–$13.43	–$2.15	6
0.67	$67.05	$14.09	0.21	$53.61	$12.11	0.23	–$13.43	–$1.98	7
0.68	$67.05	$14.09	0.21	$53.61	$12.26	0.23	–$13.43	–$1.83	7
0.69	$67.05	$14.09	0.21	$53.61	$12.41	0.23	–$13.43	–$1.68	8
0.7	$67.05	$14.09	0.21	$53.61	$12.56	0.23	–$13.43	–$1.53	9
0.71	$67.05	$14.09	0.21	$53.61	$12.69	0.24	–$13.43	–$1.40	10
0.72	$67.05	$14.09	0.21	$53.61	$12.82	0.24	–$13.43	–$1.27	11
0.73	$67.05	$14.09	0.21	$53.61	$12.95	0.24	–$13.43	–$1.14	12
0.74	$67.05	$14.09	0.21	$53.61	$13.06	0.24	–$13.43	–$1.03	13
0.75	$67.05	$14.09	0.21	$53.61	$13.17	0.25	–$13.43	–$0.92	15
0.76	$67.05	$14.09	0.21	$53.61	$13.28	0.25	–$13.43	–$0.81	17

	Option 1†			Option 2†			Relative value of option 2		
Sensitivity	Cost	Benefit	ROI‡	Cost	Benefit	ROI‡	Inc. cost§	Inc. benefit¶	Rel. ROI#
0.77	$67.05	$14.09	0.21	$53.61	$13.38	0.25	–$13.43	–$0.71	19
0.78	$67.05	$14.09	0.21	$53.61	$13.47	0.25	–$13.43	–$0.62	22
0.79	$67.05	$14.09	0.21	$53.61	$13.56	0.25	–$13.43	–$0.53	25
0.8	$67.05	$14.09	0.21	$53.61	$13.64	0.25	–$13.43	–$0.45	30
0.81	$67.05	$14.09	0.21	$53.61	$13.72	0.26	–$13.43	–$0.37	36
0.82	$67.05	$14.09	0.21	$53.61	$13.79	0.26	–$13.43	–$0.30	45
0.83	$67.05	$14.09	0.21	$53.61	$13.86	0.26	–$13.43	–$0.23	58
0.84	$67.05	$14.09	0.21	$53.61	$13.92	0.26	–$13.43	–$0.17	80

*Cost reported in USD; the exchange rate of ¥132 per $1 USD was used as the annual average in 2022 based on the report by the Bank of Japan (*4*). The model input for sensitivity of wastewater surveillance was sns_WW. Gray shading indicates ROI >1. Inc., incremental; Rel., relative.

†Option 1 is clinical tests only; option 2 is wastewater surveillance and clinical tests. Inc., incremental; ROI, return on investment. If an option is cost-saving compared with its comparator, the option's ROI is estimated to exceed 1. The comparator of options 1 and 2 is do-nothing.

‡ROI is benefit divided by cost for each option.

§Incremental cost is the cost of option 2 minus cost of option 1. A negative value of incremental cost indicates that option 2 has a lower cost or is cost-saving, compared with option 1. This could be interpreted as option 2's relative benefit.

¶Incremental benefit is the benefit of option 2 minus benefit of option 1. A negative value of incremental benefit indicates that option 2 has a lower benefit compared with option 1, which could be interpreted as option 2's relative cost.

#Relative ROI is incremental cost divided by incremental benefit.

Appendix Table 7. One-way sensitivity analysis for sensitivity of clinical PCR testing used in an economic evaluation of wastewater surveillance combined with clinical COVID-19 screening tests, Japan*

	Option 1†			Option 2†			Relative value of option 2		
Sensitivity	Cost	Benefit	ROI‡	Cost	Benefit	ROI‡	Inc. cost§	Inc. benefit¶	Rel. ROI#
0.64	$67.05	$12.36	0.18	$53.61	$11.94	0.22	–$13.43	–$0.42	32
0.65	$67.05	$12.56	0.19	$53.61	$11.94	0.22	–$13.43	–$0.62	22
0.66	$67.05	$12.76	0.19	$53.61	$11.94	0.22	–$13.43	–$0.82	16
0.67	$67.05	$12.95	0.19	$53.61	$11.94	0.22	–$13.43	–$1.01	13
0.68	$67.05	$13.13	0.20	$53.61	$11.94	0.22	–$13.43	–$1.19	11
0.69	$67.05	$13.30	0.20	$53.61	$11.94	0.22	–$13.43	–$1.36	10
0.7	$67.05	$13.47	0.20	$53.61	$11.94	0.22	–$13.43	–$1.53	9
0.71	$67.05	$13.64	0.20	$53.61	$11.94	0.22	–$13.43	–$1.70	8
0.72	$67.05	$13.79	0.21	$53.61	$11.94	0.22	–$13.43	–$1.85	7
0.73	$67.05	$13.94	0.21	$53.61	$11.94	0.22	–$13.43	–$2.00	7
0.74	$67.05	$14.09	0.21	$53.61	$11.94	0.22	–$13.43	–$2.15	6
0.75	$67.05	$14.23	0.21	$53.61	$11.94	0.22	–$13.43	–$2.29	6
0.76	$67.05	$14.37	0.21	$53.61	$11.94	0.22	–$13.43	–$2.43	6
0.77	$67.05	$14.49	0.22	$53.61	$11.94	0.22	–$13.43	–$2.56	5
0.78	$67.05	$14.62	0.22	$53.61	$11.94	0.22	–$13.43	–$2.68	5
0.79	$67.05	$14.74	0.22	$53.61	$11.94	0.22	–$13.43	–$2.80	5
0.8	$67.05	$14.85	0.22	$53.61	$11.94	0.22	–$13.43	–$2.91	5
0.81	$67.05	$14.97	0.22	$53.61	$11.94	0.22	–$13.43	–$3.03	4
0.82	$67.05	$15.07	0.22	$53.61	$11.94	0.22	–$13.43	–$3.13	4
0.83	$67.05	$15.17	0.23	$53.61	$11.94	0.22	–$13.43	–$3.23	4

*Cost reported in USD; the exchange rate of ¥132 per $1 USD was used as the annual average in 2022 based on the report by the Bank of Japan (*4*). The model input for sensitivity of PCR testing was sns_PCR. Gray shading indicates ROI >1. Inc., incremental; Rel., relative.

†Option 1 is clinical tests only; option 2 is wastewater surveillance and clinical tests. Inc., incremental; ROI, return on investment. If an option is cost-saving compared with its comparator, the option's ROI is estimated to exceed 1. The comparator of options 1 and 2 is do-nothing.

‡ROI is benefit divided by cost for each option.

§Incremental cost is the cost of option 2 minus cost of option 1. A negative value of incremental cost indicates that option 2 has a lower cost or is cost-saving, compared with option 1. This could be interpreted as option 2's relative benefit.

¶Incremental benefit is the benefit of option 2 minus benefit of option 1. A negative value of incremental benefit indicates that option 2 has a lower benefit compared with option 1, which could be interpreted as option 2's relative cost.

#Relative ROI is incremental cost divided by incremental benefit.

Appendix Table 8. One-way sensitivity analysis for the ratio of sensitivity of antigen test compared with PCR test in an economic evaluation of wastewater surveillance combined with clinical COVID-19 screening tests, Japan*

	Option 1†			Option 2†			Relative value of option 2		
Sensitivity	Cost	Benefit	ROI‡	Cost	Benefit	ROI‡	Inc. cost§	Inc. benefit¶	Rel. ROI#
0.54	$67.05	$9.47	0.14	$53.61	$11.94	0.22	–$13.43	$2.47	−5.43
0.55	$67.05	$9.76	0.15	$53.61	$11.94	0.22	–$13.43	$2.18	−6.16
0.56	$67.05	$10.04	0.15	$53.61	$11.94	0.22	–$13.43	$1.90	−7.07
0.57	$67.05	$10.31	0.15	$53.61	$11.94	0.22	–$13.43	$1.63	−8.26
0.58	$67.05	$10.58	0.16	$53.61	$11.94	0.22	–$13.43	$1.36	−9.87
0.59	$67.05	$10.84	0.16	$53.61	$11.94	0.22	–$13.43	$1.10	−12.16
0.6	$67.05	$11.08	0.17	$53.61	$11.94	0.22	–$13.43	$0.86	−15.69
0.61	$67.05	$11.32	0.17	$53.61	$11.94	0.22	–$13.43	$0.62	−21.82
0.62	$67.05	$11.56	0.17	$53.61	$11.94	0.22	–$13.43	$0.38	−35.05
0.63	$67.05	$11.78	0.18	$53.61	$11.94	0.22	–$13.43	$0.16	−84.77
0.64	$67.05	$12.00	0.18	$53.61	$11.94	0.22	–$13.43	–$0.06	228
0.65	$67.05	$12.21	0.18	$53.61	$11.94	0.22	–$13.43	–$0.27	50
0.66	$67.05	$12.41	0.19	$53.61	$11.94	0.22	–$13.43	–$0.47	28
0.67	$67.05	$12.61	0.19	$53.61	$11.94	0.22	–$13.43	–$0.67	20
0.68	$67.05	$12.80	0.19	$53.61	$11.94	0.22	–$13.43	–$0.86	16
0.69	$67.05	$12.98	0.19	$53.61	$11.94	0.22	–$13.43	–$1.04	13
0.7	$67.05	$13.16	0.20	$53.61	$11.94	0.22	–$13.43	–$1.22	11
0.71	$67.05	$13.33	0.20	$53.61	$11.94	0.22	–$13.43	–$1.39	10
0.72	$67.05	$13.49	0.20	$53.61	$11.94	0.22	–$13.43	–$1.55	9
0.73	$67.05	$13.65	0.20	$53.61	$11.94	0.22	–$13.43	–$1.71	8
0.74	$67.05	$13.80	0.21	$53.61	$11.94	0.22	–$13.43	–$1.86	7
0.75	$67.05	$13.95	0.21	$53.61	$11.94	0.22	–$13.43	–$2.01	7
0.76	$67.05	$14.09	0.21	$53.61	$11.94	0.22	–$13.43	–$2.15	6
0.77	$67.05	$14.23	0.21	$53.61	$11.94	0.22	–$13.43	–$2.29	6
0.78	$67.05	$14.36	0.21	$53.61	$11.94	0.22	–$13.43	–$2.42	6
0.79	$67.05	$14.48	0.22	$53.61	$11.94	0.22	–$13.43	–$2.55	5
0.8	$67.05	$14.61	0.22	$53.61	$11.94	0.22	–$13.43	–$2.67	5
0.81	$67.05	$14.72	0.22	$53.61	$11.94	0.22	–$13.43	–$2.78	5
0.82	$67.05	$14.84	0.22	$53.61	$11.94	0.22	–$13.43	–$2.90	5
0.83	$67.05	$14.95	0.22	$53.61	$11.94	0.22	–$13.43	–$3.01	4
0.84	$67.05	$15.05	0.22	$53.61	$11.94	0.22	–$13.43	–$3.11	4
0.85	$67.05	$15.15	0.23	$53.61	$11.94	0.22	–$13.43	–$3.21	4

	Option 1†			Option 2†			Relative value of option 2		
Sensitivity	Cost	Benefit	ROI‡	Cost	Benefit	ROI‡	Inc. cost§	Inc. benefit¶	Rel. ROI#
0.86	$67.05	$15.25	0.23	$53.61	$11.94	0.22	–$13.43	–$3.31	4
0.87	$67.05	$15.34	0.23	$53.61	$11.94	0.22	–$13.43	–$3.40	4
0.88	$67.05	$15.43	0.23	$53.61	$11.94	0.22	–$13.43	–$3.49	4
0.89	$67.05	$15.51	0.23	$53.61	$11.94	0.22	–$13.43	–$3.57	4
0.9	$67.05	$15.60	0.23	$53.61	$11.94	0.22	–$13.43	–$3.66	4
0.91	$67.05	$15.68	0.23	$53.61	$11.94	0.22	–$13.43	–$3.74	4
0.92	$67.05	$15.75	0.23	$53.61	$11.94	0.22	–$13.43	–$3.81	4
0.93	$67.05	$15.82	0.24	$53.61	$11.94	0.22	–$13.43	–$3.88	3
0.94	$67.05	$15.89	0.24	$53.61	$11.94	0.22	–$13.43	–$3.95	3
0.95	$67.05	$15.96	0.24	$53.61	$11.94	0.22	–$13.43	–$4.02	3
0.96	$67.05	$16.03	0.24	$53.61	$11.94	0.22	–$13.43	–$4.09	3
0.97	$67.05	$16.09	0.24	$53.61	$11.94	0.22	–$13.43	–$4.15	3

*Cost reported in USD; the exchange rate of ¥132 per $1 USD was used as the annual average in 2022 based on the report by the Bank of Japan (*4*). The model input for the ratio of sensitivity of antigen test against PCR test was sns_Ag_ratio_PCR. Gray shading indicates ROI >1. Inc., incremental; Rel., relative.

†Option 1 is clinical tests only; option 2 is wastewater surveillance and clinical tests. Inc., incremental; ROI, return on investment. If an option is cost-saving compared with its comparator, the option's ROI is estimated to exceed 1. The comparator of options 1 and 2 is do-nothing.

‡ROI is benefit divided by cost for each option.

§Incremental cost is the cost of option 2 minus cost of option 1. A negative value of incremental cost indicates that option 2 has a lower cost or is cost-saving, compared with option 1. This could be interpreted as option 2's relative benefit.

¶Incremental benefit is the benefit of option 2 minus benefit of option 1. A negative value of incremental benefit indicates that option 2 has a lower benefit compared with option 1, which could be interpreted as option 2's relative cost.

#Relative ROI is incremental cost divided by incremental benefit.

Appendix Table 9. One-way sensitivity analysis for sensitivity of clinical PCR test subsequent to a positive antigen test in an economic evaluation of wastewater surveillance combined with clinical COVID-19 screening tests, Japan*

	Option 1†			Option 2†			Relative value of option 2		
Sensitivity	Cost	Benefit	ROI‡	Cost	Benefit	ROI‡	Inc. cost§	Inc. benefit¶	Rel. ROI#
0.64	$67.06	$7.91	0.12	$53.62	$5.39	0.10	–$13.44	–$2.52	5
0.7	$67.05	$9.39	0.14	$53.62	$6.93	0.13	–$13.44	–$2.46	5
0.8	$67.05	$11.42	0.17	$53.62	$9.09	0.17	–$13.43	–$2.33	6
0.9	$67.05	$13.00	0.19	$53.62	$10.77	0.20	–$13.43	–$2.22	6
0.99	$67.05	$14.09	0.21	$53.61	$11.94	0.22	–$13.43	–$2.15	6
0.999	$67.05	$14.19	0.21	$53.61	$12.04	0.22	–$13.43	–$2.14	6

*Cost reported in USD; the exchange rate of ¥132 per $1 USD was used as the annual average in 2022 based on the report by the Bank of Japan (*4*). The model input for sensitivity of PCR test subsequent to a positive antigen test was sns_PCR_2nd. Gray shading indicates ROI >1. Inc., incremental; Rel., relative.

†Option 1 is clinical tests only; option 2 is wastewater surveillance and clinical tests. Inc., incremental; ROI, return on investment. If an option is cost-saving compared with its comparator, the option's ROI is estimated to exceed 1. The comparator of options 1 and 2 is do-nothing.

‡ROI is benefit divided by cost for each option.

§Incremental cost is the cost of option 2 minus cost of option 1. A negative value of incremental cost indicates that option 2 has a lower cost or is cost-saving, compared with option 1. This could be interpreted as option 2's relative benefit.

¶Incremental benefit is the benefit of option 2 minus benefit of option 1. A negative value of incremental benefit indicates that option 2 has a lower benefit compared with option 1, which could be interpreted as option 2's relative cost.

#Relative ROI is incremental cost divided by incremental benefit.

Appendix Table 10. One-way sensitivity analysis for specificity of clinical PCR test used in an economic evaluation of wastewater surveillance combined with clinical COVID-19 screening tests, Japan*

	Option 1†			Option 2†			Relative value of option 2		
Specificity	Cost	Benefit	ROI‡	Cost	Benefit	ROI‡	Inc. cost§	Inc. benefit¶	Rel. ROI#
0.960	$67.05	$14.06	0.21	$56.77	$11.80	0.21	–$10.27	–$2.26	5
0.961	$67.05	$14.06	0.21	$56.55	$11.82	0.21	–$10.50	–$2.25	5
0.962	$67.05	$14.06	0.21	$56.32	$11.83	0.21	–$10.73	–$2.24	5
0.963	$67.05	$14.07	0.21	$56.10	$11.84	0.21	–$10.95	–$2.23	5
0.964	$67.05	$14.07	0.21	$55.87	$11.85	0.21	–$11.18	–$2.22	5
0.965	$67.05	$14.07	0.21	$55.64	$11.86	0.21	–$11.40	–$2.21	5
0.966	$67.05	$14.07	0.21	$55.42	$11.87	0.21	–$11.63	–$2.20	5
0.967	$67.05	$14.08	0.21	$55.19	$11.88	0.22	–$11.85	–$2.19	5
0.968	$67.05	$14.08	0.21	$54.97	$11.89	0.22	–$12.08	–$2.19	6
0.969	$67.05	$14.08	0.21	$54.74	$11.90	0.22	–$12.31	–$2.18	6
0.97	$67.05	$14.08	0.21	$54.52	$11.91	0.22	–$12.53	–$2.17	6
0.971	$67.05	$14.08	0.21	$54.29	$11.92	0.22	–$12.76	–$2.17	6
0.972	$67.05	$14.09	0.21	$54.07	$11.93	0.22	–$12.98	–$2.16	6
0.973	$67.05	$14.09	0.21	$53.84	$11.93	0.22	–$13.21	–$2.16	6
0.974	$67.05	$14.09	0.21	$53.61	$11.94	0.22	–$13.43	–$2.15	6
0.975	$67.05	$14.09	0.21	$53.39	$11.95	0.22	–$13.66	–$2.15	6
0.976	$67.05	$14.09	0.21	$53.16	$11.95	0.22	–$13.88	–$2.14	6
0.977	$67.05	$14.10	0.21	$52.94	$11.96	0.23	–$14.11	–$2.14	7
0.978	$67.05	$14.10	0.21	$52.71	$11.96	0.23	–$14.34	–$2.13	7
0.979	$67.05	$14.10	0.21	$52.49	$11.97	0.23	–$14.56	–$2.13	7
0.98	$67.05	$14.10	0.21	$52.26	$11.97	0.23	–$14.79	–$2.13	7
0.981	$67.05	$14.10	0.21	$52.03	$11.97	0.23	–$15.01	–$2.13	7
0.982	$67.05	$14.10	0.21	$51.81	$11.98	0.23	–$15.24	–$2.12	7
0.983	$67.05	$14.10	0.21	$51.58	$11.98	0.23	–$15.46	–$2.12	7
0.984	$67.05	$14.10	0.21	$51.36	$11.98	0.23	–$15.69	–$2.12	7
0.985	$67.05	$14.11	0.21	$51.13	$11.99	0.23	–$15.92	–$2.12	8
0.986	$67.05	$14.11	0.21	$50.91	$11.99	0.24	–$16.14	–$2.12	8
0.987	$67.05	$14.11	0.21	$50.68	$11.99	0.24	–$16.37	–$2.12	8
0.988	$67.05	$14.11	0.21	$50.45	$11.99	0.24	–$16.59	–$2.12	8
0.989	$67.05	$14.11	0.21	$50.23	$11.99	0.24	–$16.82	–$2.11	8
0.99	$67.05	$14.11	0.21	$50.00	$12.00	0.24	–$17.04	–$2.11	8
0.991	$67.05	$14.11	0.21	$49.78	$12.00	0.24	–$17.27	–$2.11	8

Specificity	Option 1† Cost	Benefit	ROI‡	Option 2† Cost	Benefit	ROI‡	Relative value of option 2 Inc. cost§	Inc. benefit¶	Rel. ROI#
0.992	$67.05	$14.11	0.21	$49.55	$12.00	0.24	–$17.50	–$2.11	8
0.993	$67.05	$14.11	0.21	$49.33	$12.00	0.24	–$17.72	–$2.11	8
0.994	$67.05	$14.11	0.21	$49.10	$12.00	0.24	–$17.95	–$2.11	8
0.995	$67.05	$14.11	0.21	$48.88	$12.00	0.25	–$18.17	–$2.11	9

*Cost reported in USD; the exchange rate of ¥132 per $1 USD was used as the annual average in 2022 based on the report by the Bank of Japan (*4*). The model input for specificity of clinical PCR test was spc_PCR. Gray shading indicates ROI >1. Inc., incremental; Rel., relative.

†Option 1 is clinical tests only; option 2 is wastewater surveillance and clinical tests. Inc., incremental; ROI, return on investment. If an option is cost-saving compared with its comparator, the option's ROI is estimated to exceed 1. The comparator of options 1 and 2 is do-nothing.

‡ROI is benefit divided by cost for each option.

§Incremental cost is the cost of option 2 minus cost of option 1. A negative value of incremental cost indicates that option 2 has a lower cost or is cost-saving, compared with option 1. This could be interpreted as option 2's relative benefit.

¶Incremental benefit is the benefit of option 2 minus benefit of option 1. A negative value of incremental benefit indicates that option 2 has a lower benefit compared with option 1, which could be interpreted as option 2's relative cost.

#Relative ROI is incremental cost divided by incremental benefit.

Appendix Table 11. One-way sensitivity analysis for specificity of antigen test used in an economic evaluation of wastewater surveillance combined with clinical COVID-19 screening tests, Japan*

Specificity	Option 1† Cost	Benefit	ROI‡	Option 2† Cost	Benefit	ROI‡	Relative value of option 2 Inc. cost§	Inc. benefit¶	Rel. ROI#
0.97	$73.12	$14.05	0.19	$53.61	$11.94	0.22	–$19.51	–$2.11	9
0.971	$72.82	$14.05	0.19	$53.61	$11.94	0.22	–$19.21	–$2.11	9
0.972	$72.52	$14.05	0.19	$53.61	$11.94	0.22	–$18.90	–$2.11	9
0.973	$72.21	$14.05	0.19	$53.61	$11.94	0.22	–$18.60	–$2.11	9
0.974	$71.91	$14.05	0.20	$53.61	$11.94	0.22	–$18.30	–$2.11	9
0.975	$71.61	$14.06	0.20	$53.61	$11.94	0.22	–$17.99	–$2.12	9
0.976	$71.30	$14.06	0.20	$53.61	$11.94	0.22	–$17.69	–$2.12	8
0.977	$71.00	$14.06	0.20	$53.61	$11.94	0.22	–$17.38	–$2.12	8
0.978	$70.69	$14.06	0.20	$53.61	$11.94	0.22	–$17.08	–$2.12	8
0.979	$70.39	$14.07	0.20	$53.61	$11.94	0.22	–$16.78	–$2.13	8
0.98	$70.09	$14.07	0.20	$53.61	$11.94	0.22	–$16.47	–$2.13	8
0.981	$69.78	$14.07	0.20	$53.61	$11.94	0.22	–$16.17	–$2.13	8
0.982	$69.48	$14.07	0.20	$53.61	$11.94	0.22	–$15.86	–$2.13	7

	Option 1†			Option 2†			Relative value of option 2		
Specificity	Cost	Benefit	ROI‡	Cost	Benefit	ROI‡	Inc. cost§	Inc. benefit¶	Rel. ROI#
0.983	$69.17	$14.07	0.20	$53.61	$11.94	0.22	–$15.56	–$2.13	7
0.984	$68.87	$14.08	0.20	$53.61	$11.94	0.22	–$15.26	–$2.14	7
0.985	$68.57	$14.08	0.21	$53.61	$11.94	0.22	–$14.95	–$2.14	7
0.986	$68.26	$14.08	0.21	$53.61	$11.94	0.22	–$14.65	–$2.14	7
0.987	$67.96	$14.08	0.21	$53.61	$11.94	0.22	–$14.35	–$2.14	7
0.988	$67.66	$14.09	0.21	$53.61	$11.94	0.22	–$14.04	–$2.15	7
0.989	$67.35	$14.09	0.21	$53.61	$11.94	0.22	–$13.74	–$2.15	6
0.99	$67.05	$14.09	0.21	$53.61	$11.94	0.22	–$13.43	–$2.15	6
0.991	$66.74	$14.09	0.21	$53.61	$11.94	0.22	–$13.13	–$2.15	6
0.992	$66.44	$14.09	0.21	$53.61	$11.94	0.22	–$12.83	–$2.15	6
0.993	$66.14	$14.10	0.21	$53.61	$11.94	0.22	–$12.52	–$2.16	6
0.994	$65.83	$14.10	0.21	$53.61	$11.94	0.22	–$12.22	–$2.16	6
0.995	$65.53	$14.10	0.22	$53.61	$11.94	0.22	–$11.91	–$2.16	6

*Cost reported in USD; the exchange rate of ¥132 per $1 USD was used as the annual average in 2022 based on the report by the Bank of Japan (*4*). The model input for specificity of antigen tests was spc_Ag. Gray shading indicates ROI >1. Inc., incremental; Rel., relative.

†Option 1 is clinical tests only; option 2 is wastewater surveillance and clinical tests. Inc., incremental; ROI, return on investment. If an option is cost-saving compared with its comparator, the option's ROI is estimated to exceed 1. The comparator of options 1 and 2 is do-nothing.

‡ROI is benefit divided by cost for each option.

§Incremental cost is the cost of option 2 minus cost of option 1. A negative value of incremental cost indicates that option 2 has a lower cost or is cost-saving, compared with option 1. This could be interpreted as option 2's relative benefit.

¶Incremental benefit is the benefit of option 2 minus benefit of option 1. A negative value of incremental benefit indicates that option 2 has a lower benefit compared with option 1, which could be interpreted as option 2's relative cost.

#Relative ROI is incremental cost divided by incremental benefit.

Appendix Table 12. One-way sensitivity analysis of laboratory cost of wastewater surveillance per day per facility in an economic evaluation of wastewater surveillance combined with clinical COVID-19 screening tests, Japan*

	Option 1†			Option 2†			Relative value of option 2		
Laboratory cost	Cost	Benefit	ROI‡	Cost	Benefit	ROI‡	Inc. cost§	Inc. benefit¶	Rel. ROI#
$189	$67.05	$14.09	0.21	$47.91	$11.94	0.25	–$19.13	–$2.15	9
$246	$67.05	$14.09	0.21	$49.62	$11.94	0.24	–$17.43	–$2.15	8
$303	$67.05	$14.09	0.21	$51.33	$11.94	0.23	–$15.72	–$2.15	7
$360	$67.05	$14.09	0.21	$53.03	$11.94	0.23	–$14.01	–$2.15	7
$417	$67.05	$14.09	0.21	$54.74	$11.94	0.22	–$12.31	–$2.15	6
$474	$67.05	$14.09	0.21	$56.45	$11.94	0.21	–$10.60	–$2.15	5
$530	$67.05	$14.09	0.21	$58.16	$11.94	0.21	–$8.89	–$2.15	4
$587	$67.05	$14.09	0.21	$59.86	$11.94	0.20	–$7.18	–$2.15	3
$644	$67.05	$14.09	0.21	$61.57	$11.94	0.19	–$5.48	–$2.15	3
$701	$67.05	$14.09	0.21	$63.28	$11.94	0.19	–$3.77	–$2.15	2
$750	$67.05	$14.09	0.21	$64.74	$11.94	0.18	–$2.30	–$2.15	1.07
$755	$67.05	$14.09	0.21	$64.89	$11.94	0.18	–$2.15	–$2.15	1.00
$756	$67.05	$14.09	0.21	$64.92	$11.94	0.18	–$2.12	–$2.15	0.99
$758	$67.05	$14.09	0.21	$64.98	$11.94	0.18	–$2.06	–$2.15	0.96

*Cost per facility per day reported in USD; the exchange rate of ¥132 per $1 USD was used as the annual average in 2022 based on the report by the Bank of Japan (*4*). The model input for laboratory cost of wastewater surveillance was C_ww_unit_per_test. Gray shading indicates ROI >1. Inc., incremental; Rel., relative.

†Option 1 is clinical tests only; option 2 is wastewater surveillance and clinical tests. Inc., incremental; ROI, return on investment. If an option is cost-saving compared with its comparator, the option's ROI is estimated to exceed 1. The comparator of options 1 and 2 is do-nothing.

‡ROI is benefit divided by cost for each option.

§Incremental cost is the cost of option 2 minus cost of option 1. A negative value of incremental cost indicates that option 2 has a lower cost or is cost-saving, compared with option 1. This could be interpreted as option 2's relative benefit.

¶Incremental benefit is the benefit of option 2 minus benefit of option 1. A negative value of incremental benefit indicates that option 2 has a lower benefit compared with option 1, which could be interpreted as option 2's relative cost.

#Relative ROI is incremental cost divided by incremental benefit.

Appendix Table 13. One-way sensitivity analysis of labor cost to sample wastewater per day per facility in an economic evaluation of wastewater surveillance combined with clinical COVID-19 screening tests, Japan*

	Option 1†			Option 2†			Relative value of option 2		
Labor cost	Cost	Benefit	ROI‡	Cost	Benefit	ROI‡	Inc. cost§	Inc. benefit¶	Rel. ROI#
$152	$67.05	$14.09	0.21	$24.09	$11.94	0.50	–$42.95	–$2.15	20

	Option 1†			Option 2†			Relative value of option 2		
Labor cost	Cost	Benefit	ROI‡	Cost	Benefit	ROI‡	Inc. cost§	Inc. benefit¶	Rel. ROI#
$341	$67.05	$14.09	0.21	$29.77	$11.94	0.40	–$37.27	–$2.15	17
$531	$67.05	$14.09	0.21	$35.45	$11.94	0.34	–$31.60	–$2.15	15
$720	$67.05	$14.09	0.21	$41.13	$11.94	0.29	–$25.92	–$2.15	12
$909	$67.05	$14.09	0.21	$46.81	$11.94	0.26	–$20.24	–$2.15	9
$1,099	$67.05	$14.09	0.21	$52.49	$11.94	0.23	–$14.56	–$2.15	7
$1,288	$67.05	$14.09	0.21	$58.17	$11.94	0.21	–$8.88	–$2.15	4
$1,477	$67.05	$14.09	0.21	$63.85	$11.94	0.19	–$3.20	–$2.15	1.49
$1,510	$67.05	$14.09	0.21	$64.83	$11.94	0.18	–$2.21	–$2.15	1.03
$1,515	$67.05	$14.09	0.21	$64.98	$11.94	0.18	–$2.06	–$2.15	0.96
$1,580	$67.05	$14.09	0.21	$66.93	$11.94	0.18	–$0.11	–$2.15	0.05
$1,585	$67.05	$14.09	0.21	$67.08	$11.94	0.18	$0.04	–$2.15	−0.02
$1,666	$67.05	$14.09	0.21	$69.53	$11.94	0.17	$2.48	–$2.15	−1.15
$1,856	$67.05	$14.09	0.21	$75.20	$11.94	0.16	$8.16	–$2.15	−3.79
$2,045	$67.05	$14.09	0.21	$80.88	$11.94	0.15	$13.84	–$2.15	−6.43

*Cost per facility per day reported in USD; the exchange rate of ¥132 per $1 USD was used as the annual average in 2022 based on the report by the Bank of Japan (*4*). The model input for labor cost of wastewater surveillance was C_ww_f_lbr. Gray shading indicates ROI >1. Inc., incremental; Rel., relative.

†Option 1 is clinical tests only; option 2 is wastewater surveillance and clinical tests. Inc., incremental; ROI, return on investment. If an option is cost-saving compared with its comparator, the option's ROI is estimated to exceed 1. The comparator of options 1 and 2 is do-nothing.

‡ROI is benefit divided by cost for each option.

§Incremental cost is the cost of option 2 minus cost of option 1. A negative value of incremental cost indicates that option 2 has a lower cost or is cost-saving, compared with option 1. This could be interpreted as option 2's relative benefit.

¶Incremental benefit is the benefit of option 2 minus benefit of option 1. A negative value of incremental benefit indicates that option 2 has a lower benefit compared with option 1, which could be interpreted as option 2's relative cost.

#Relative ROI is incremental cost divided by incremental benefit.

Appendix Table 14. One-way sensitivity analysis of cost for antigen test in an economic evaluation of wastewater surveillance combined with clinical COVID-19 screening tests, Japan*

Antigen test	Option 1†			Option 2†			Relative value of option 2		
cost	Cost	Benefit	ROI‡	Cost	Benefit	ROI‡	Inc. cost§	Inc. benefit¶	Rel. ROI#
$10	$43.05	$14.09	0.33	$53.61	$11.94	0.22	$10.56	–$2.15	−4.91
$11	$47.05	$14.09	0.30	$53.61	$11.94	0.22	$6.56	–$2.15	−3.05
$12	$51.05	$14.09	0.28	$53.61	$11.94	0.22	$2.56	–$2.15	−1.19

Antigen test	Option 1†			Option 2†			Relative value of option 2		
cost	Cost	Benefit	ROI‡	Cost	Benefit	ROI‡	Inc. cost§	Inc. benefit¶	Rel. ROI#
$13	$55.05	$14.09	0.26	$53.61	$11.94	0.22	–$1.44	–$2.15	0.67
$13.18	$55.77	$14.09	0.25	$53.61	$11.94	0.22	–$2.16	–$2.15	1.00
$14	$59.05	$14.09	0.24	$53.61	$11.94	0.22	–$5.43	–$2.15	3
$15	$63.05	$14.09	0.22	$53.61	$11.94	0.22	–$9.43	–$2.15	4
$16	$67.05	$14.09	0.21	$53.61	$11.94	0.22	–$13.43	–$2.15	6
$17	$71.05	$14.09	0.20	$53.61	$11.94	0.22	–$17.43	–$2.15	8
$18	$75.05	$14.09	0.19	$53.61	$11.94	0.22	–$21.43	–$2.15	10
$19	$79.05	$14.09	0.18	$53.61	$11.94	0.22	–$25.43	–$2.15	12
$20	$83.05	$14.09	0.17	$53.61	$11.94	0.22	–$29.43	–$2.15	14
$21	$87.04	$14.09	0.16	$53.61	$11.94	0.22	–$33.43	–$2.15	16
$22	$91.04	$14.09	0.15	$53.61	$11.94	0.22	–$37.43	–$2.15	17
$23	$95.04	$14.09	0.15	$53.61	$11.94	0.22	–$41.43	–$2.15	19

*Cost reported in USD; the exchange rate of ¥132 per $1 USD was used as the annual average in 2022 based on the report by the Bank of Japan (*4*). The model input for antigen test cost was C_Ag. Gray shading indicates ROI >1. Inc., incremental; Rel., relative.

†Option 1 is clinical tests only; option 2 is wastewater surveillance and clinical tests. Inc., incremental; ROI, return on investment. If an option is cost-saving compared with its comparator, the option's ROI is estimated to exceed 1. The comparator of options 1 and 2 is do-nothing.

‡ROI is benefit divided by cost for each option.

§Incremental cost is the cost of option 2 minus cost of option 1. A negative value of incremental cost indicates that option 2 has a lower cost or is cost-saving, compared with option 1. This could be interpreted as option 2's relative benefit.

¶Incremental benefit is the benefit of option 2 minus benefit of option 1. A negative value of incremental benefit indicates that option 2 has a lower benefit compared with option 1, which could be interpreted as option 2's relative cost.

#Relative ROI is incremental cost divided by incremental benefit.

Appendix Table 15. One-way sensitivity analysis of cost for clinical PCR tests in an economic evaluation of wastewater surveillance combined with clinical COVID-19 screening tests, Japan*

Clinical	Option 1†			Option 2†			Relative value of option 2		
PCR test cost	Cost	Benefit	ROI‡	Cost	Benefit	ROI‡	Inc. cost§	Inc. benefit¶	Rel. ROI#
$20	$65.60	$14.08	0.215	$49.75	$11.94	0.24	–$15.85	–$2.15	7
$23	$65.86	$14.09	0.214	$50.46	$11.94	0.24	–$15.41	–$2.15	7
$27	$66.13	$14.09	0.213	$51.16	$11.94	0.23	–$14.97	–$2.15	7
$30	$66.40	$14.09	0.212	$51.87	$11.94	0.23	–$14.52	–$2.15	7
$33	$66.66	$14.09	0.211	$52.58	$11.94	0.23	–$14.08	–$2.15	7
$37	$66.93	$14.09	0.211	$53.29	$11.94	0.22	–$13.64	–$2.15	6

Clinical PCR test cost	Option 1†			Option 2†			Relative value of option 2		
	Cost	Benefit	ROI‡	Cost	Benefit	ROI‡	Inc. cost§	Inc. benefit¶	Rel. ROI#
$40	$67.19	$14.09	0.210	$54.00	$11.94	0.22	–$13.19	–$2.15	6
$43	$67.46	$14.09	0.209	$54.71	$11.94	0.22	–$12.75	–$2.15	6
$46	$67.72	$14.09	0.208	$55.42	$11.94	0.22	–$12.30	–$2.15	6
$50	$67.99	$14.09	0.207	$56.13	$11.94	0.21	–$11.86	–$2.15	6
$53	$68.25	$14.10	0.207	$56.84	$11.94	0.21	–$11.42	–$2.15	5

*Cost reported in USD; the exchange rate of ¥132 per $1 USD was used as the annual average in 2022 based on the report by the Bank of Japan (*4*). The model input for clinical PCR test cost was C_PCR. Gray shading indicates ROI >1. Inc., incremental; Rel., relative.

†Option 1 is clinical tests only; option 2 is wastewater surveillance and clinical tests. Inc., incremental; ROI, return on investment. If an option is cost-saving compared with its comparator, the option's ROI is estimated to exceed 1. The comparator of options 1 and 2 is do-nothing.

‡ROI is benefit divided by cost for each option.

§Incremental cost is the cost of option 2 minus cost of option 1. A negative value of incremental cost indicates that option 2 has a lower cost or is cost-saving, compared with option 1. This could be interpreted as option 2's relative benefit.

¶Incremental benefit is the benefit of option 2 minus benefit of option 1. A negative value of incremental benefit indicates that option 2 has a lower benefit compared with option 1, which could be interpreted as option 2's relative cost.

#Relative ROI is incremental cost divided by incremental benefit.

Appendix Table 16. One-way sensitivity analysis of cost for isolation per case in an economic evaluation of wastewater surveillance combined with clinical COVID-19 screening tests, Japan*

	Option 1†			Option 2†			Relative value of option 2		
Isolation cost	Cost	Benefit	ROI‡	Cost	Benefit	ROI‡	Inc. cost§	Inc. benefit¶	Rel. ROI#
$379	$67.05	$14.03	0.209	$53.61	$11.90	0.222	–$13.43	–$2.12	6.33
$455	$67.05	$14.04	0.209	$53.61	$11.91	0.222	–$13.43	–$2.13	6.31
$530	$67.05	$14.05	0.210	$53.61	$11.92	0.222	–$13.43	–$2.13	6.30
$606	$67.05	$14.06	0.210	$53.61	$11.93	0.222	–$13.43	–$2.14	6.28
$682	$67.05	$14.08	0.210	$53.61	$11.93	0.223	–$13.43	–$2.14	6.26
$758	$67.05	$14.09	0.210	$53.61	$11.94	0.223	–$13.43	–$2.15	6.25
$833	$67.05	$14.10	0.210	$53.61	$11.95	0.223	–$13.43	–$2.16	6.23
$909	$67.05	$14.12	0.211	$53.61	$11.95	0.223	–$13.43	–$2.16	6.21
$985	$67.05	$14.13	0.211	$53.61	$11.96	0.223	–$13.43	–$2.17	6.20
$1,061	$67.05	$14.14	0.211	$53.61	$11.97	0.223	–$13.43	–$2.17	6.18
$1,136	$67.05	$14.15	0.211	$53.61	$11.98	0.223	–$13.43	–$2.18	6.17
$1,212	$67.05	$14.17	0.211	$53.61	$11.98	0.224	–$13.43	–$2.18	6.15
$1,288	$67.05	$14.18	0.212	$53.61	$11.99	0.224	–$13.43	–$2.19	6.13
$1,364	$67.05	$14.19	0.212	$53.61	$12.00	0.224	–$13.43	–$2.20	6.12
$1,439	$67.05	$14.21	0.212	$53.61	$12.01	0.224	–$13.43	–$2.20	6.10
$1,515	$67.05	$14.22	0.212	$53.61	$12.01	0.224	–$13.43	–$2.21	6.09

*Cost reported in USD; the exchange rate of ¥132 per $1 USD was used as the annual average in 2022 based on the report by the Bank of Japan (*4*). The model input for cost for isolation was C_isolation. Gray shading indicates ROI >1. Inc., incremental; Rel., relative.

†Option 1 is clinical tests only; option 2 is wastewater surveillance and clinical tests. Inc., incremental; ROI, return on investment. If an option is cost-saving compared with its comparator, the option's ROI is estimated to exceed 1. The comparator of options 1 and 2 is do-nothing.

‡ROI is benefit divided by cost for each option.

§Incremental cost is the cost of option 2 minus cost of option 1. A negative value of incremental cost indicates that option 2 has a lower cost or is cost-saving, compared with option 1. This could be interpreted as option 2's relative benefit.

¶Incremental benefit is the benefit of option 2 minus benefit of option 1. A negative value of incremental benefit indicates that option 2 has a lower benefit compared with option 1, which could be interpreted as option 2's relative cost.

#Relative ROI is incremental cost divided by incremental benefit.

Appendix Table 17. One-way sensitivity analysis of hospitalization cost per case in an economic evaluation of wastewater surveillance combined with clinical COVID-19 screening tests, Japan*

Hospitalization	Option 1†			Option 2†			Relative value of option 2		
cost	Cost	Benefit	ROI‡	Cost	Benefit	ROI‡	Inc. cost§	Inc. benefit¶	Rel. ROI#
$16,212	$67.05	$12.30	0.18	$53.61	$10.42	0.19	–$13.43	–$1.88	7
$16,856	$67.05	$12.66	0.19	$53.61	$10.72	0.20	–$13.43	–$1.94	7
$17,500	$67.05	$13.02	0.19	$53.61	$11.03	0.21	–$13.43	–$1.99	7
$18,144	$67.05	$13.38	0.20	$53.61	$11.33	0.21	–$13.43	–$2.04	7
$18,788	$67.05	$13.73	0.20	$53.61	$11.64	0.22	–$13.43	–$2.10	6
$19,432	$67.05	$14.09	0.21	$53.61	$11.94	0.22	–$13.43	–$2.15	6
$20,076	$67.05	$14.45	0.22	$53.61	$12.24	0.23	–$13.43	–$2.20	6
$20,720	$67.05	$14.80	0.22	$53.61	$12.55	0.23	–$13.43	–$2.26	6
$21,363	$67.05	$15.16	0.23	$53.61	$12.85	0.24	–$13.43	–$2.31	6
$22,007	$67.05	$15.52	0.23	$53.61	$13.15	0.25	–$13.43	–$2.36	6
$22,651	$67.05	$15.88	0.24	$53.61	$13.46	0.25	–$13.43	–$2.42	6
$23,295	$67.05	$16.23	0.24	$53.61	$13.76	0.26	–$13.43	–$2.47	5
$23,939	$67.05	$16.59	0.25	$53.61	$14.07	0.26	–$13.43	–$2.52	5
$24,583	$67.05	$16.95	0.25	$53.61	$14.37	0.27	–$13.43	–$2.58	5
$25,227	$67.05	$17.31	0.26	$53.61	$14.67	0.27	–$13.43	–$2.63	5

*Cost reported in USD; the exchange rate of ¥132 per $1 USD was used as the annual average in 2022 based on the report by the Bank of Japan (*4*). The model input for cost for hospitalization was C_hosp_all. Gray shading indicates ROI >1. Inc., incremental; Rel., relative.

†Option 1 is clinical tests only; option 2 is wastewater surveillance and clinical tests. Inc., incremental; ROI, return on investment. If an option is cost-saving compared with its comparator, the option's ROI is estimated to exceed 1. The comparator of options 1 and 2 is do-nothing.

‡ROI is benefit divided by cost for each option.

§Incremental cost is the cost of option 2 minus cost of option 1. A negative value of incremental cost indicates that option 2 has a lower cost or is cost-saving, compared with option 1. This could be interpreted as option 2's relative benefit.

¶Incremental benefit is the benefit of option 2 minus benefit of option 1. A negative value of incremental benefit indicates that option 2 has a lower benefit compared with option 1, which could be interpreted as option 2's relative cost.

#Relative ROI is incremental cost divided by incremental benefit.

Appendix Table 18. One-way sensitivity analysis of COVID-19 incidence per million population in an economic evaluation of wastewater surveillance combined with clinical screening tests, Japan*

	Option 1†			Option 2†			Relative value of option 2		
Incidence	Cost	Benefit	ROI‡	Cost	Benefit	ROI‡	Inc. cost§	Inc. benefit¶	Rel. ROI#
10	$67.04	$1.39	0.02	$53.60	$1.14	0.02	–$13.44	–$0.25	54
50	$67.04	$7.03	0.10	$53.61	$5.94	0.11	–$13.44	–$1.09	12
100	$67.05	$14.09	0.21	$53.61	$11.94	0.22	–$13.43	–$2.15	6
400	$67.07	$56.43	0.84	$53.66	$47.94	0.89	–$13.41	–$8.49	1.58
445	$67.08	$62.78	0.94	$53.67	$53.34	0.99	–$13.41	–$9.44	1.42
450	$67.08	$63.49	0.95	$53.67	$53.94	1.01	–$13.41	–$9.54	1.40
475	$67.08	$67.01	0.999	$53.67	$56.94	1.06	–$13.41	–$10.07	1.33
480	$67.08	$67.72	1.010	$53.67	$57.54	1.07	–$13.41	–$10.18	1.32
500	$67.08	$70.54	1.05	$53.68	$59.94	1.12	–$13.40	–$10.60	1.26
600	$67.09	$84.65	1.26	$53.69	$71.94	1.34	–$13.40	–$12.71	1.05
630	$67.09	$88.89	1.32	$53.70	$75.54	1.41	–$13.39	–$13.35	1.004
635	$67.09	$89.59	1.34	$53.70	$76.14	1.42	–$13.39	–$13.45	0.996
700	$67.10	$98.77	1.47	$53.71	$83.94	1.56	–$13.39	–$14.83	0.90
1,000	$67.12	$141.11	2	$53.75	$119.94	2	–$13.37	–$21.16	0.63
2,000	$67.20	$282.23	4	$53.91	$239.95	4	–$13.29	–$42.29	0.31
5,000	$67.45	$705.62	10	$54.37	$599.96	11	–$13.07	–$105.66	0.12
10,000	$67.85	$1,411.26	21	$55.14	$1,199.97	22	–$12.71	–$211.29	0.06

*Cost reported in USD; the exchange rate of ¥132 per $1 USD was used as the annual average in 2022 based on the report by the Bank of Japan (*4*). The model input for incidence was inc_n_per_M. Gray shading indicates ROI >1. Inc., incremental; Rel., relative.

†Option 1 is clinical tests only; option 2 is wastewater surveillance and clinical tests. Inc., incremental; ROI, return on investment. If an option is cost-saving compared with its comparator, the option's ROI is estimated to exceed 1. The comparator of options 1 and 2 is do-nothing.

‡ROI is benefit divided by cost for each option.

§Incremental cost is the cost of option 2 minus cost of option 1. A negative value of incremental cost indicates that option 2 has a lower cost or is cost-saving, compared with option 1. This could be interpreted as option 2's relative benefit.

¶Incremental benefit is the benefit of option 2 minus benefit of option 1. A negative value of incremental benefit indicates that option 2 has a lower benefit compared with option 1, which could be interpreted as option 2's relative cost.

#Relative ROI is incremental cost divided by incremental benefit.

Appendix Table 19. One-way sensitivity analysis of number of residents at a facility in an economic evaluation of wastewater surveillance combined with COVID-19 clinical screening tests, Japan*

No. residents	Option 1†			Option 2†			Relative value of option 2		
at a facility	Cost	Benefit	ROI‡	Cost	Benefit	ROI‡	Inc. cost§	Inc. benefit¶	Rel. ROI#
50	$67.05	$14.09	0.21	$99.06	$11.94	0.12	$32.02	–$2.15	−14.89
60	$67.05	$14.09	0.21	$83.91	$11.94	0.14	$16.87	–$2.15	−7.84
70	$67.05	$14.09	0.21	$73.09	$11.94	0.16	$6.05	–$2.15	−2.81
80	$67.05	$14.09	0.21	$64.98	$11.94	0.18	–$2.07	–$2.15	0.96
81	$67.05	$14.09	0.21	$64.28	$11.94	0.19	–$2.77	–$2.15	1.29
90	$67.05	$14.09	0.21	$58.66	$11.94	0.20	–$8.38	–$2.15	4
100	$67.05	$14.09	0.21	$53.61	$11.94	0.22	–$13.43	–$2.15	6
200	$67.05	$14.09	0.21	$30.89	$11.94	0.39	–$36.16	–$2.15	17
300	$67.05	$14.09	0.21	$23.31	$11.94	0.51	–$43.73	–$2.15	20
400	$67.05	$14.09	0.21	$19.53	$11.94	0.61	–$47.52	–$2.15	22
500	$67.05	$14.09	0.21	$17.25	$11.94	0.69	–$49.79	–$2.15	23
600	$67.05	$14.09	0.21	$15.74	$11.94	0.76	–$51.31	–$2.15	24
700	$67.05	$14.09	0.21	$14.66	$11.94	0.81	–$52.39	–$2.15	24
800	$67.05	$14.09	0.21	$13.85	$11.94	0.86	–$53.20	–$2.15	25
900	$67.05	$14.09	0.21	$13.21	$11.94	0.90	–$53.83	–$2.15	25
1,000	$67.05	$14.09	0.21	$12.71	$11.94	0.94	–$54.34	–$2.15	25

*Cost reported in USD; the exchange rate of ¥132 per $1 USD was used as the annual average in 2022 based on the report by the Bank of Japan (*4*). The model input for number of residents was N_facility_size. Gray shading indicates ROI >1. Inc., incremental; Rel., relative.

†Option 1 is clinical tests only; option 2 is wastewater surveillance and clinical tests. Inc., incremental; ROI, return on investment. If an option is cost-saving compared with its comparator, the option's ROI is estimated to exceed 1. The comparator of options 1 and 2 is do-nothing.

‡ROI is benefit divided by cost for each option.

§Incremental cost is the cost of option 2 minus cost of option 1. A negative value of incremental cost indicates that option 2 has a lower cost or is cost-saving, compared with option 1. This could be interpreted as option 2's relative benefit.

¶Incremental benefit is the benefit of option 2 minus benefit of option 1. A negative value of incremental benefit indicates that option 2 has a lower benefit compared with option 1, which could be interpreted as option 2's relative cost.

#Relative ROI is incremental cost divided by incremental benefit.

Appendix Table 20. One-way sensitivity analysis of mortality rate among persons who tested COVID-19 positive in an economic evaluation of wastewater surveillance combined with clinical screening tests, Japan*

	Option 1†			Option 2†			Relative value of option 2		
Mortality rate	Cost	Benefit	ROI‡	Cost	Benefit	ROI‡	Inc. cost§	Inc. benefit¶	Rel. ROI#
0.0018	$67.05	$12.55	0.19	$53.61	$10.63	0.20	–$13.43	–$1.92	7
0.002	$67.05	$12.73	0.19	$53.61	$10.79	0.20	–$13.43	–$1.95	7
0.003	$67.05	$13.64	0.20	$53.61	$11.56	0.22	–$13.43	–$2.08	6
0.004	$67.05	$14.54	0.22	$53.61	$12.32	0.23	–$13.43	–$2.22	6
0.005	$67.05	$15.45	0.23	$53.61	$13.09	0.24	–$13.43	–$2.35	6
0.006	$67.05	$16.35	0.24	$53.61	$13.86	0.26	–$13.43	–$2.49	5
0.007	$67.05	$17.26	0.26	$53.61	$14.63	0.27	–$13.43	–$2.62	5
0.008	$67.05	$18.16	0.27	$53.61	$15.40	0.29	–$13.43	–$2.76	5
0.009	$67.05	$19.07	0.28	$53.61	$16.17	0.30	–$13.43	–$2.90	5
0.0104	$67.05	$20.33	0.30	$53.61	$17.25	0.32	–$13.43	–$3.09	4

*Cost reported in USD; the exchange rate of ¥132 per $1 USD was used as the annual average in 2022 based on the report by the Bank of Japan (*4*). The model input for the mortality rate was r_mortality_test_positive. Gray shading indicates ROI >1. Inc., incremental; Rel., relative.

†Option 1 is clinical tests only; option 2 is wastewater surveillance and clinical tests. Inc., incremental; ROI, return on investment. If an option is cost-saving compared with its comparator, the option's ROI is estimated to exceed 1. The comparator of options 1 and 2 is do-nothing.

‡ROI is benefit divided by cost for each option.

§Incremental cost is the cost of option 2 minus cost of option 1. A negative value of incremental cost indicates that option 2 has a lower cost or is cost-saving, compared with option 1. This could be interpreted as option 2's relative benefit.

¶Incremental benefit is the benefit of option 2 minus benefit of option 1. A negative value of incremental benefit indicates that option 2 has a lower benefit compared with option 1, which could be interpreted as option 2's relative cost.

#Relative ROI is incremental cost divided by incremental benefit.

Appendix Table 21. One-way sensitivity analysis of the ratio of mortality rates among persons >80 years of age compared with the general population in an economic evaluation of wastewater surveillance combined with COVID-19 clinical screening tests, Japan*

	Option 1†			Option 2†			Relative value of option 2		
Ratio	Cost	Benefit	ROI‡	Cost	Benefit	ROI‡	Inc. cost§	Inc. benefit¶	Rel. ROI#
0.5	$67.05	$0.28	0.004	$53.61	$0.20	0.004	–$13.43	–$0.08	161
0.6	$67.05	$0.36	0.005	$53.61	$0.26	0.005	–$13.43	–$0.09	142
0.7	$67.05	$0.43	0.006	$53.61	$0.33	0.006	–$13.43	–$0.11	127
0.8	$67.05	$0.51	0.008	$53.61	$0.39	0.007	–$13.43	–$0.12	115
0.9	$67.05	$0.58	0.009	$53.61	$0.45	0.008	–$13.43	–$0.13	105
1	$67.05	$0.66	0.010	$53.61	$0.52	0.010	–$13.43	–$0.14	96
1.1	$67.05	$0.73	0.011	$53.61	$0.58	0.011	–$13.43	–$0.15	89
1.2	$67.05	$0.80	0.012	$53.61	$0.64	0.012	–$13.43	–$0.16	83
1.3	$67.05	$0.88	0.013	$53.61	$0.71	0.013	–$13.43	–$0.17	78
1.4	$67.05	$0.95	0.014	$53.61	$0.77	0.014	–$13.43	–$0.18	73
1.5	$67.05	$1.03	0.02	$53.61	$0.83	0.02	–$13.43	–$0.20	69
15	$67.05	$11.10	0.17	$53.61	$9.40	0.18	–$13.43	–$1.70	8
16	$67.05	$11.85	0.18	$53.61	$10.04	0.19	–$13.43	–$1.82	7
17	$67.05	$12.60	0.19	$53.61	$10.67	0.20	–$13.43	–$1.93	7
18	$67.05	$13.34	0.20	$53.61	$11.31	0.21	–$13.43	–$2.04	7
19	$67.05	$14.09	0.21	$53.61	$11.94	0.22	–$13.43	–$2.15	6
20	$67.05	$14.84	0.22	$53.61	$12.57	0.23	–$13.43	–$2.26	6
21	$67.05	$15.58	0.23	$53.61	$13.21	0.25	–$13.43	–$2.37	6
22	$67.05	$16.33	0.24	$53.61	$13.84	0.26	–$13.43	–$2.49	5

*Cost reported in USD; the exchange rate of ¥132 per $1 USD was used as the annual average in 2022 based on the report by the Bank of Japan (*4*). The model input for the ratio of mortality rates among persons >80 years of age compared with persons <80 years of age was V_mr_ratio. Gray shading indicates ROI >1. Inc., incremental; Rel., relative.

†Option 1 is clinical tests only; option 2 is wastewater surveillance and clinical tests. Inc., incremental; ROI, return on investment. If an option is cost-saving compared with its comparator, the option's ROI is estimated to exceed 1. The comparator of options 1 and 2 is do-nothing.

‡ROI is benefit divided by cost for each option.

§Incremental cost is the cost of option 2 minus cost of option 1. A negative value of incremental cost indicates that option 2 has a lower cost or is cost-saving, compared with option 1. This could be interpreted as option 2's relative benefit.

¶Incremental benefit is the benefit of option 2 minus benefit of option 1. A negative value of incremental benefit indicates that option 2 has a lower benefit compared with option 1, which could be interpreted as option 2's relative cost.

#Relative ROI is incremental cost divided by incremental benefit.

Appendix Table 22. One-way sensitivity analysis of life years saved due to avoiding COVID-19 infection in an economic evaluation of wastewater surveillance combined with COVID-19 clinical screening tests, Japan*

	Option 1†			Option 2†			Relative value of option 2		
Years saved	Cost	Benefit	ROI‡	Cost	Benefit	ROI‡	Inc. cost§	Inc. benefit¶	Rel. ROI#
11.1	$67.05	$14.01	0.209	$53.61	$11.87	0.221	–$13.43	–$2.14	6.28
11.2	$67.05	$14.03	0.209	$53.61	$11.89	0.222	–$13.43	–$2.14	6.27
11.3	$67.05	$14.06	0.210	$53.61	$11.92	0.222	–$13.43	–$2.15	6.26
11.4	$67.05	$14.09	0.210	$53.61	$11.94	0.223	–$13.43	–$2.15	6.25
11.5	$67.05	$14.12	0.211	$53.61	$11.96	0.223	–$13.43	–$2.15	6.23
11.6	$67.05	$14.15	0.211	$53.61	$11.99	0.224	–$13.43	–$2.16	6.22
11.7	$67.05	$14.17	0.211	$53.61	$12.01	0.224	–$13.43	–$2.16	6.21

*Cost reported in USD; the exchange rate of ¥132 per $1 USD was used as the annual average in 2022 based on the report by the Bank of Japan (*4*). The model input for life years saved was V_life_yrs_saved. Gray shading indicates ROI >1. Inc., incremental; Rel., relative.

†Option 1 is clinical tests only; option 2 is wastewater surveillance and clinical tests. Inc., incremental; ROI, return on investment. If an option is cost-saving compared with its comparator, the option's ROI is estimated to exceed 1. The comparator of options 1 and 2 is do-nothing.

‡ROI is benefit divided by cost for each option.

§Incremental cost is the cost of option 2 minus cost of option 1. A negative value of incremental cost indicates that option 2 has a lower cost or is cost-saving, compared with option 1. This could be interpreted as option 2's relative benefit.

¶Incremental benefit is the benefit of option 2 minus benefit of option 1. A negative value of incremental benefit indicates that option 2 has a lower benefit compared with option 1, which could be interpreted as option 2's relative cost.

#Relative ROI is incremental cost divided by incremental benefit.

Appendix Table 23. One-way sensitivity analysis of the ratio to convert life-years saved to quality adjusted life years (QALYs) saved in an economic evaluation of wastewater surveillance combined with COVID-19 clinical screening tests, Japan*

	Option 1†			Option 2†			Relative value of option 2		
Ratio	Cost	Benefit	ROI‡	Cost	Benefit	ROI‡	Inc. cost§	Inc. benefit¶	Rel. ROI#
0.64	$67.05	$13.90	0.207	$53.61	$11.78	0.220	–$13.43	–$2.12	6.33
0.65	$67.05	$13.95	0.208	$53.61	$11.82	0.220	–$13.43	–$2.13	6.31
0.66	$67.05	$14.00	0.209	$53.61	$11.86	0.221	–$13.43	–$2.14	6.29
0.67	$67.05	$14.04	0.209	$53.61	$11.90	0.222	–$13.43	–$2.14	6.27
0.68	$67.05	$14.09	0.210	$53.61	$11.94	0.223	–$13.43	–$2.15	6.25
0.69	$67.05	$14.14	0.211	$53.61	$11.98	0.223	–$13.43	–$2.16	6.23
0.7	$67.05	$14.18	0.212	$53.61	$12.02	0.224	–$13.43	–$2.16	6.21
0.71	$67.05	$14.23	0.212	$53.61	$12.06	0.225	–$13.43	–$2.17	6.19

*Cost reported in USD; the exchange rate of ¥132 per $1 USD was used as the annual average in 2022 based on the report by the Bank of Japan (*4*). The model input for the ratio to convert life-years saved to quality adjusted life years was r_QALY_adj. Gray shading indicates ROI >1. Inc., incremental; Rel., relative.

†Option 1 is clinical tests only; option 2 is wastewater surveillance and clinical tests. Inc., incremental; ROI, return on investment. If an option is cost-saving compared with its comparator, the option's ROI is estimated to exceed 1. The comparator of options 1 and 2 is do-nothing.

‡ROI is benefit divided by cost for each option.

§Incremental cost is the cost of option 2 minus cost of option 1. A negative value of incremental cost indicates that option 2 has a lower cost or is cost-saving, compared with option 1. This could be interpreted as option 2's relative benefit.

¶Incremental benefit is the benefit of option 2 minus benefit of option 1. A negative value of incremental benefit indicates that option 2 has a lower benefit compared with option 1, which could be interpreted as option 2's relative cost.

#Relative ROI is incremental cost divided by incremental benefit.

Appendix Table 24. One-way sensitivity analysis of hospitalization rates among persons who tested COVID-19 positive in an economic evaluation of wastewater surveillance combined with COVID-19 clinical screening tests, Japan*

	Option 1†			Option 2†			Relative value of option 2		
Rate	Cost	Benefit	ROI‡	Cost	Benefit	ROI‡	Inc. cost§	Inc. benefit¶	Rel. ROI#
0.04	$67.05	$5.41	0.08	$53.61	$4.46	0.08	–$13.43	–$0.95	14
0.06	$67.05	$6.63	0.10	$53.61	$5.50	0.10	–$13.43	–$1.14	12
0.08	$67.05	$7.86	0.12	$53.61	$6.54	0.12	–$13.43	–$1.32	10
0.1	$67.05	$9.09	0.14	$53.61	$7.60	0.14	–$13.43	–$1.49	9
0.12	$67.05	$10.33	0.15	$53.61	$8.67	0.16	–$13.43	–$1.66	8
0.14	$67.05	$11.58	0.17	$53.61	$9.75	0.18	–$13.43	–$1.83	7
0.16	$67.05	$12.83	0.19	$53.61	$10.84	0.20	–$13.43	–$1.99	7
0.18	$67.05	$14.09	0.21	$53.61	$11.94	0.22	–$13.43	–$2.15	6
0.2	$67.05	$15.35	0.23	$53.61	$13.05	0.24	–$13.43	–$2.30	6
0.22	$67.05	$16.62	0.25	$53.61	$14.17	0.26	–$13.43	–$2.45	5
0.24	$67.05	$17.89	0.27	$53.61	$15.29	0.29	–$13.43	–$2.60	5
0.26	$67.05	$19.17	0.29	$53.61	$16.43	0.31	–$13.43	–$2.74	5
0.28	$67.05	$20.45	0.31	$53.61	$17.57	0.33	–$13.43	–$2.88	5
0.3	$67.05	$21.74	0.32	$53.61	$18.72	0.35	–$13.43	–$3.02	4
0.32	$67.05	$23.03	0.34	$53.61	$19.88	0.37	–$13.43	–$3.15	4
0.34	$67.05	$24.32	0.36	$53.61	$21.04	0.39	–$13.43	–$3.28	4
0.36	$67.05	$25.62	0.38	$53.61	$22.21	0.41	–$13.43	–$3.41	4
0.38	$67.05	$26.92	0.40	$53.61	$23.39	0.44	–$13.43	–$3.53	4
0.40	$67.05	$28.23	0.42	$53.61	$24.57	0.46	–$13.43	–$3.66	4

*Cost reported in USD; the exchange rate of ¥132 per $1 USD was used as the annual average in 2022 based on the report by the Bank of Japan (*4*). The model input for the hospitalization rates among persons who tested COVID-19 positive was r_hosp_test_positive. Gray shading indicates ROI >1. Inc., incremental; Rel., relative.

†Option 1 is clinical tests only; option 2 is wastewater surveillance and clinical tests. Inc., incremental; ROI, return on investment. If an option is cost-saving compared with its comparator, the option's ROI is estimated to exceed 1. The comparator of options 1 and 2 is do-nothing.

‡ROI is benefit divided by cost for each option.

§Incremental cost is the cost of option 2 minus cost of option 1. A negative value of incremental cost indicates that option 2 has a lower cost or is cost-saving, compared with option 1. This could be interpreted as option 2's relative benefit.

¶Incremental benefit is the benefit of option 2 minus benefit of option 1. A negative value of incremental benefit indicates that option 2 has a lower benefit compared with option 1, which could be interpreted as option 2's relative cost.

#Relative ROI is incremental cost divided by incremental benefit.

Appendix Table 25. One-way sensitivity analysis for the proportion of severe cases among hospitalized cases in an economic evaluation of wastewater surveillance combined with COVID-19 clinical screening tests, Japan*

	Option 1†			Option 2†			Relative value of option 2		
Proportion	Cost	Benefit	ROI‡	Cost	Benefit	ROI‡	Inc. cost§	Inc. benefit¶	Rel. ROI#
0.05	$67.05	$12.28	0.18	$53.61	$10.38	0.19	–$13.43	–$1.90	7
0.06	$67.05	$12.64	0.19	$53.61	$10.69	0.20	–$13.43	–$1.95	7
0.07	$67.05	$13.00	0.19	$53.61	$11.00	0.21	–$13.43	–$2.00	7
0.08	$67.05	$13.37	0.20	$53.61	$11.31	0.21	–$13.43	–$2.05	7
0.09	$67.05	$13.73	0.20	$53.61	$11.63	0.22	–$13.43	–$2.10	6
0.1	$67.05	$14.09	0.21	$53.61	$11.94	0.22	–$13.43	–$2.15	6
0.11	$67.05	$14.45	0.22	$53.61	$12.25	0.23	–$13.43	–$2.20	6
0.12	$67.05	$14.82	0.22	$53.61	$12.57	0.23	–$13.43	–$2.25	6
0.13	$67.05	$15.18	0.23	$53.61	$12.88	0.24	–$13.43	–$2.30	6
0.14	$67.05	$15.54	0.23	$53.61	$13.20	0.25	–$13.43	–$2.35	6
0.15	$67.05	$15.91	0.24	$53.61	$13.51	0.25	–$13.43	–$2.40	6
0.16	$67.05	$16.27	0.24	$53.61	$13.83	0.26	–$13.43	–$2.44	5
0.17	$67.05	$16.64	0.25	$53.61	$14.14	0.26	–$13.43	–$2.49	5
0.18	$67.05	$17.00	0.25	$53.61	$14.46	0.27	–$13.43	–$2.54	5
0.19	$67.05	$17.36	0.26	$53.61	$14.78	0.28	–$13.43	–$2.59	5

*Cost reported in USD; the exchange rate of ¥132 per $1 USD was used as the annual average in 2022 based on the report by the Bank of Japan (*4*). The model input for the proportion of severe cases was r_icu_hosp. Gray shading indicates ROI >1. Inc., incremental; Rel., relative.

†Option 1 is clinical tests only; option 2 is wastewater surveillance and clinical tests. Inc., incremental; ROI, return on investment. If an option is cost-saving compared with its comparator, the option's ROI is estimated to exceed 1. The comparator of options 1 and 2 is do-nothing.

‡ROI is benefit divided by cost for each option.

§Incremental cost is the cost of option 2 minus cost of option 1. A negative value of incremental cost indicates that option 2 has a lower cost or is cost-saving, compared with option 1. This could be interpreted as option 2's relative benefit.

¶Incremental benefit is the benefit of option 2 minus benefit of option 1. A negative value of incremental benefit indicates that option 2 has a lower benefit compared with option 1, which could be interpreted as option 2's relative cost.

#Relative ROI is incremental cost divided by incremental benefit.

Appendix Table 26. One-way sensitivity analysis of effective reproduction number (R_e) in an economic evaluation of wastewater surveillance combined with COVID-19 clinical screening tests, Japan*

	Option 1†			Option 2†			Relative value of option 2		
R_e	Cost	Benefit	ROI‡	Cost	Benefit	ROI‡	Inc. cost§	Inc. benefit¶	Rel. ROI#
0.9	$67.05	$18.63	0.28	$53.62	$16.70	0.31	–$13.43	–$1.93	7

	Option 1†			Option 2†			Relative value of option 2		
R_e	Cost	Benefit	ROI‡	Cost	Benefit	ROI‡	Inc. cost§	Inc. benefit¶	Rel. ROI#
1	$67.05	$17.02	0.25	$53.62	$15.05	0.28	–$13.43	–$1.97	7
1.1	$67.05	$15.80	0.24	$53.62	$13.78	0.26	–$13.43	–$2.02	7
1.2	$67.05	$14.85	0.22	$53.62	$12.77	0.24	–$13.43	–$2.08	6
1.3	$67.05	$14.09	0.21	$53.61	$11.94	0.22	–$13.43	–$2.15	6
1.4	$67.05	$13.48	0.20	$53.61	$11.25	0.21	–$13.43	–$2.23	6
1.5	$67.05	$12.98	0.19	$53.61	$10.66	0.20	–$13.43	–$2.32	6
1.6	$67.05	$12.56	0.19	$53.61	$10.15	0.19	–$13.43	–$2.41	6
1.7	$67.05	$12.21	0.18	$53.61	$9.70	0.18	–$13.43	–$2.51	5
1.8	$67.05	$11.92	0.18	$53.61	$9.30	0.17	–$13.44	–$2.62	5
1.9	$67.05	$11.66	0.17	$53.61	$8.93	0.17	–$13.44	–$2.73	5
2.0	$67.05	$11.44	0.17	$53.61	$8.60	0.16	–$13.44	–$2.84	5

*Cost reported in USD; the exchange rate of ¥132 per $1 USD was used as the annual average in 2022 based on the report by the Bank of Japan (*4*). The model input for effective reproduction number was R_e. Gray shading indicates ROI >1. Inc., incremental; R_e, effective reproduction number; Rel., relative.

†Option 1 is clinical tests only; option 2 is wastewater surveillance and clinical tests. Inc., incremental; ROI, return on investment. If an option is cost-saving compared with its comparator, the option's ROI is estimated to exceed 1. The comparator of options 1 and 2 is do-nothing.

‡ROI is benefit divided by cost for each option.

§Incremental cost is the cost of option 2 minus cost of option 1. A negative value of incremental cost indicates that option 2 has a lower cost or is cost-saving, compared with option 1. This could be interpreted as option 2's relative benefit.

¶Incremental benefit is the benefit of option 2 minus benefit of option 1. A negative value of incremental benefit indicates that option 2 has a lower benefit compared with option 1, which could be interpreted as option 2's relative cost.

#Relative ROI is incremental cost divided by incremental benefit.

Appendix Table 27. One-way sensitivity analysis of effectiveness of screening in reducing hospitalization and mortality rates because of an earlier COVID-19 diagnosis in an economic evaluation of wastewater surveillance combined with clinical screening tests, Japan*

	Option 1†			Option 2†			Relative value of option 2		
Effectiveness	Cost	Benefit	ROI‡	Cost	Benefit	ROI‡	Inc. cost§	Inc. benefit¶	Rel. ROI#
0.23	$67.05	$6.08	0.09	$53.61	$5.13	0.10	–$13.43	–$0.95	14
0.25	$67.05	$6.60	0.10	$53.61	$5.57	0.10	–$13.43	–$1.03	13
0.3	$67.05	$7.89	0.12	$53.61	$6.67	0.12	–$13.43	–$1.22	11
0.35	$67.05	$9.18	0.14	$53.61	$7.77	0.14	–$13.43	–$1.42	9
0.4	$67.05	$10.47	0.16	$53.61	$8.87	0.17	–$13.43	–$1.61	8
0.45	$67.05	$11.77	0.18	$53.61	$9.96	0.19	–$13.43	–$1.80	7
0.5	$67.05	$13.06	0.19	$53.61	$11.06	0.21	–$13.43	–$2.00	7
0.55	$67.05	$14.35	0.21	$53.61	$12.16	0.23	–$13.43	–$2.19	6
0.6	$67.05	$15.64	0.23	$53.61	$13.26	0.25	–$13.43	–$2.38	6
0.62	$67.05	$16.16	0.24	$53.61	$13.70	0.26	–$13.43	–$2.46	5

*Cost reported in USD; the exchange rate of ¥132 per $1 USD was used as the annual average in 2022 based on the report by the Bank of Japan (*4*). The model input for effectiveness of early diagnosis was Eff_early_Dx. Gray shading indicates ROI >1. Inc., incremental; Rel., relative.

†Option 1 is clinical tests only; option 2 is wastewater surveillance and clinical tests. Inc., incremental; ROI, return on investment. If an option is cost-saving compared with its comparator, the option's ROI is estimated to exceed 1. The comparator of options 1 and 2 is do-nothing.

‡ROI is benefit divided by cost for each option.

§Incremental cost is the cost of option 2 minus cost of option 1. A negative value of incremental cost indicates that option 2 has a lower cost or is cost-saving, compared with option 1. This could be interpreted as option 2's relative benefit.

¶Incremental benefit is the benefit of option 2 minus benefit of option 1. A negative value of incremental benefit indicates that option 2 has a lower benefit compared with option 1, which could be interpreted as option 2's relative cost.

#Relative ROI is incremental cost divided by incremental benefit.

Appendix Table 28. One-way sensitivity analysis of the ratio of the loss value of missing an infected case compared with the benefit value of finding an infected case in an economic evaluation of wastewater surveillance combined with COVID-19 clinical screening tests, Japan*

Ratio of	Option 1†			Option 2†			Relative value of option 2		
loss value	Cost	Benefit	ROI‡	Cost	Benefit	ROI‡	Inc. cost§	Inc. benefit¶	Rel. ROI#
0.0	$67.05	$16.74	0.25	$53.61	$16.65	0.31	–$13.43	–$0.09	148
0.1	$67.05	$16.48	0.25	$53.61	$16.18	0.30	–$13.43	–$0.30	45
0.2	$67.05	$16.21	0.24	$53.61	$15.71	0.29	–$13.43	–$0.50	27
0.3	$67.05	$15.95	0.24	$53.61	$15.24	0.28	–$13.43	–$0.71	19
0.4	$67.05	$15.68	0.23	$53.61	$14.77	0.28	–$13.43	–$0.91	15
0.5	$67.05	$15.42	0.23	$53.61	$14.30	0.27	–$13.43	–$1.12	12
0.6	$67.05	$15.15	0.23	$53.61	$13.82	0.26	–$13.43	–$1.33	10
0.7	$67.05	$14.89	0.22	$53.61	$13.35	0.25	–$13.43	–$1.53	9
0.8	$67.05	$14.62	0.22	$53.61	$12.88	0.24	–$13.43	–$1.74	8
0.9	$67.05	$14.36	0.21	$53.61	$12.41	0.23	–$13.43	–$1.94	7
1	$67.05	$14.09	0.21	$53.61	$11.94	0.22	–$13.43	–$2.15	6
1.1	$67.05	$13.83	0.21	$53.61	$11.47	0.21	–$13.43	–$2.36	6
1.2	$67.05	$13.56	0.20	$53.61	$11.00	0.21	–$13.43	–$2.56	5
1.3	$67.05	$13.29	0.20	$53.61	$10.53	0.20	–$13.43	–$2.77	5
1.4	$67.05	$13.03	0.19	$53.61	$10.06	0.19	–$13.43	–$2.97	5
1.5	$67.05	$12.76	0.19	$53.61	$9.58	0.18	–$13.43	–$3.18	4
1.6	$67.05	$12.50	0.19	$53.61	$9.11	0.17	–$13.43	–$3.39	4
1.7	$67.05	$12.23	0.18	$53.61	$8.64	0.16	–$13.43	–$3.59	4
1.8	$67.05	$11.97	0.18	$53.61	$8.17	0.15	–$13.43	–$3.80	4
1.9	$67.05	$11.70	0.17	$53.61	$7.70	0.14	–$13.43	–$4.00	3
2.0	$67.05	$11.44	0.17	$53.61	$7.23	0.13	–$13.43	–$4.21	3

*Cost reported in USD; the exchange rate of ¥132 per $1 USD was used as the annual average in 2022 based on the report by the Bank of Japan (*4*). The model input for the ratio of the loss value was r_B_Loss. Gray shading indicates ROI >1. Inc., incremental; Rel., relative.

†Option 1 is clinical tests only; option 2 is wastewater surveillance and clinical tests. Inc., incremental; ROI, return on investment. If an option is cost-saving compared with its comparator, the option's ROI is estimated to exceed 1. The comparator of options 1 and 2 is do-nothing.

‡ROI is benefit divided by cost for each option.

§Incremental cost is the cost of option 2 minus cost of option 1. A negative value of incremental cost indicates that option 2 has a lower cost or is cost-saving, compared with option 1. This could be interpreted as option 2's relative benefit.

¶Incremental benefit is the benefit of option 2 minus benefit of option 1. A negative value of incremental benefit indicates that option 2 has a lower benefit compared with option 1, which could be interpreted as option 2's relative cost.

#Relative ROI is incremental cost divided by incremental benefit.

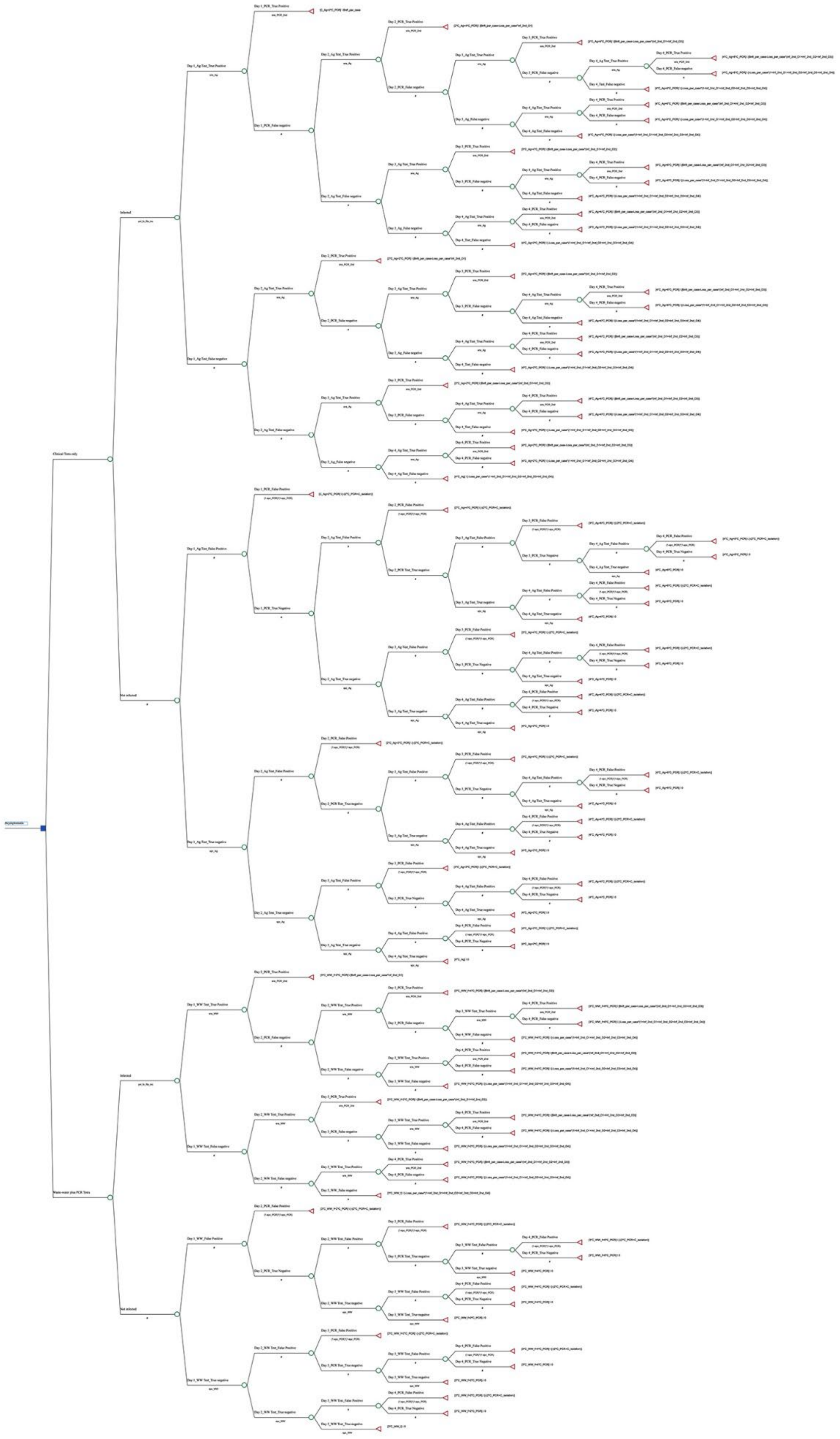

Appendix Figure 1. Decision tree used in an economic evaluation of wastewater surveillance combined with COVID-19 clinical screening tests, Japan. A blue square indicates a choice facing the decision maker. Green circles indicate an event that has multiple possible outcomes and is not under the decision maker's control; red triangles indicate the endpoint of a scenario. Bnft_per_case, benefit of finding one infected case by a screening; Loss_per_case, value of missing one infected case; C_Ag, antigen test cost; C_hosp_all, hospitalization cost per case; C_isolation, isolation cost for a test-positive; C_PCR, clinical PCR test cost; C_ww_f, labor cost (to sample at a facility) plus laboratory cost of wastewater surveillance per day per facility; Inf_2nd_D1, the number of newly produced second-generation infected cases on Day 1; Inf_2nd_D2, the number of newly produced second-generation infected cases on Day 2; Inf_2nd_D3, the number of newly produced second-generation infected cases on Day 3; Inf_2nd_D4, the number of newly produced second-generation infected cases on Day 4; sns_Ag, sensitivity of antigen test; sns_PCR_2nd, sensitivity of clinical PCR test subsequent to a positive test of antigen test; sns_WW, sensitivity of wastewater surveillance; spc_Ag, specificity of antigen test; spc_PCR, specificity of clinical PCR test.

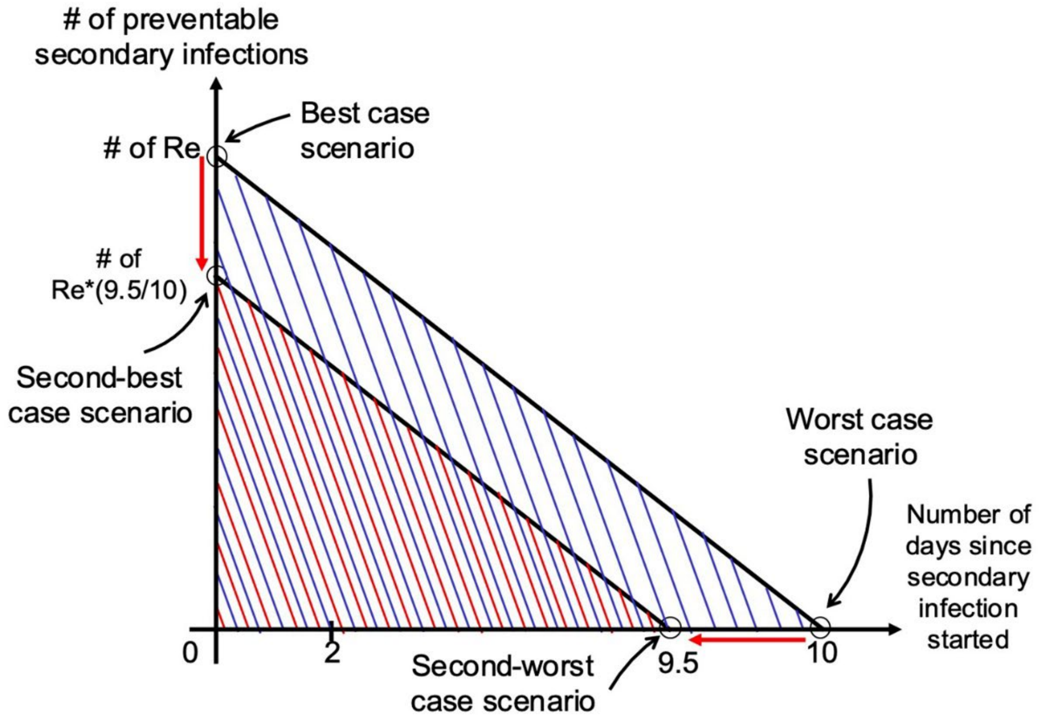

Note: Re represents reproduction number.

Appendix Figure 2. Number of preventable secondary infections assumed in an economic evaluation of wastewater surveillance combined with COVID-19 clinical screening tests, Japan. Y-axis indicates the number of preventable secondary infections for each day during an infectious period, which is indicated by x-axis. Blue striped (lighter shadow in black and white) area indicates the probabilistic summed number of the preventable secondary infections based on the less realistic best-case and worst-case scenarios explained in the text. Red and blue striped (darker shadow in black and white) area indicates the probabilistic summed number of the preventable secondary infections based on the more realistic second-best-case and second-worst-case scenarios.

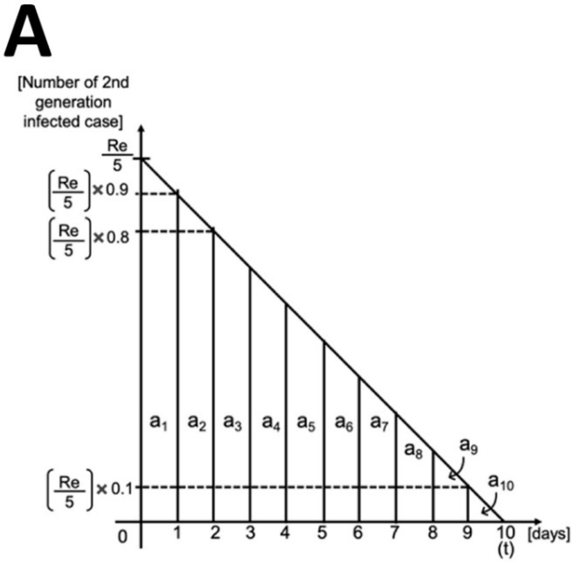

Appendix Figure 3. Number of second-generation infected cases yielded by a missed first-generation infected case in an economic evaluation of wastewater surveillance combined with COVID-19 clinical screening tests, Japan. A) Assumed 10-day infectious period; B) Infectious period is expressed as t, and was assigned a triangular distribution. A trapezoid area “a_i” indicates the number of second-generation infected cases yielded by a missed first-generation infected case for day “i” during an infectious period, which is indicated by X-axis. Only for the final day of the infectious period, a far-right triangular area (a_{10} in A and a_t in B) indicates the number of second-generation infected cases yielded by a missed first-generation infected case; R_e, effective reproduction number; t, time in days during an infectious period.

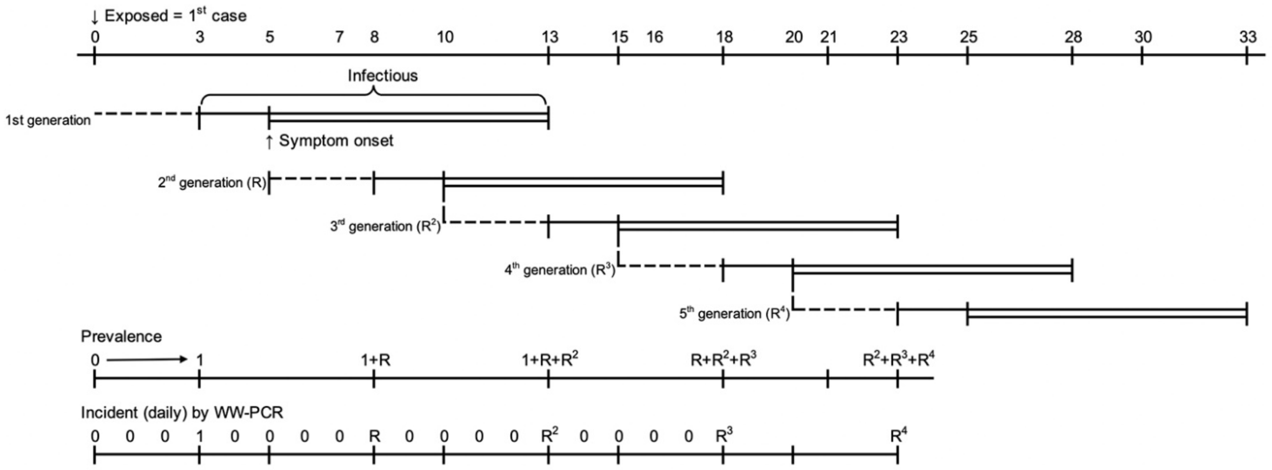

Appendix Figure 4. Relationship between prevalence and incidence in an economic evaluation of wastewater surveillance combined with COVID-19 clinical screening tests, Japan. The timeline demonstrates time in days from the first case being infected over several generations. Each subsequent generation is assumed to be produced only on the symptom onset day of a previous generation for simplicity. The bottom 2 lines indicate prevalence and daily incidence. A part of daily incidence (newly infected cases) is detected by wastewater surveillance (WW) or antigen tests, subsequently confirmed by clinical PCR tests.
